# Spirit of the English Periodicals, and Notices of English Medical Literature

**Published:** 1836-01-01

**Authors:** 


### Periscope;

OR,

### CIRCUMSPECTIVE REVIEW.

" Ore trahit quodcunque potest, atque addit acervo."

I.

### Spirit of the English Periodicals, and Notices of English Medical Literature.

FATAL ABDOMINAL DISEASE OF INSIDIOUS ORIGIN. By JAMES JOHNSON, M.D.

A YOUNG lady, aged 23 years, of active habits and cheerful disposition, had occasionally complained, for nearly two years past, of some pain in the left side of the abdomen; and for the last six months, she felt enlarged in that region, which she attributed to fat, and bewailed this precocious corpulency. During the greater part of September, 1835, she had been with her grandmother in the country, whence she returned to town, on Saturday the 19th of September, in as good health and spirits as she had ever enjoyed in her life. On the succeeding Tuesday (Sept. 22d) she was seized with pain in the abdomen, and some sickness at stomach, for which she took a dose of castor oil. She was not much relieved, and next morning (Wednesday) she sent for Mr. Hunter, of Hart-street, Bloomsbury, who found her with obstructed bowels, tenderness and tension of the abdomen, and nausea. She was leeched—calomel and opium were exhibited, and purgatives were given. Mr. Hunter declares that plentiful fæcal evacuations were procured on the Thursday and Friday —after which nothing like fæculent matter was passed, till Tuesday the 29th of September, when Dr. Johnson was called in. The abdomen was now much enlarged, tense, tender, and the greater part of it, especially in the inferior and left side, was solid on percussion. The superior parts were tympanitic. Nausea and vomiting were frequent, the pulse was quick, and the countenance dejected. Leeches were freely applied to the abdomen—fomentations—repeated injections—and small doses of calomel, with effervescing draughts, were administered. On Wednesday (30th) she was bled from the arm, till a tendency to syncope took place. The blood was highly inflamed. No passage could be procured through the bowels by any medicine or enema. On Thursday the 1st of October, however, there were twelve or fourteen evacuations, decidedly fæculent, and it was hoped by some of the medical attendants (for there were now three or four) that as the obstruction had given way, there was some prospect of recovery. The enlargement and tension of the abdomen remained unabated—sickness continued—and prostration of strength was on the increase. She died at seven o'clock on Friday morning, October the second.

*Examination.* At half-past seven in the evening of the day she died, the body was examined by Mr. Henry James Johnson, in presence of Mr. Hunter, Mr. Andrews, Dr. Johnson, and another gentleman. On opening the abdomen, the omentum was found adhering, in several places, to the peritoneum, and the whole of the latter membrane was sprinkled with melanotic deposits, varying in size, from a small kidney-bean to a millet seed. While separating these outward adhesions, a sac was penetrated, which discharged three or four pints of thin, fætid, seropurulent matter. This sac was very irregular and extensive, occupying the left hypochondrium and even the pelvis of that side. Its parietes were lined with a very thick layer of coagulable lymph, of old standing, being, in many places, as thick as a penny-piece. A great many convolutions of the intestines were glued together—some by old and unlacerable adhesions—others by recent exudations of coagulable lymph, that were easily torn. The intestinal tube, in many places, was so doubled upon itself, and bound down by adhesions, at such acute angles, that peristaltic motion, in those portions, was almost impossible; and it is difficult to imagine how the fæcal remains could have found their way through such agglutinated tubes! It could only be by the *vis a tergo*, for very little contractile power could have been exerted in the tube itself, in these localities. Between several folds of the small intestines, there were depôts of sero-purulent matter, contained in sacs formed of the original exudations of lymph. Excepting a small quantity of liquid fæces in the colon, there were no accumulations in the bowels. No ulceration of the mucous membrane—no volvulus or intus-susception.

The case is remarkable, as shewing the extent of disease which may obtain, with little apparent disturbance of the general health. Twelve days before this young lady's death, and when three or four pints of sero-purulent fluid existed in the abdomen, she appeared in excellent health and spirits, running up and down stairs, and taking an active part in all domestic arrangements, in consequence of her mother being confined to her room by ill health! The only thing she complained of, and that in rather a jocose manner, was the increase of corpulency; which attracted the notice of the family and friends. The inflammation of which she died, was acute supervening on chronic—a dangerous occurrence at all times. This case illustrates another circumstance which many must have observed, namely, the free evacuation of fæces, after a long and obstinate inflammatory obstruction, and yet without affording any substantial relief. Young practitioners are thus thrown off their guard, and promise recovery to the friends, while the old and experienced attendant suspends his prognosis till he sees whether the fever, sickness, and other symptoms subside, with the evacuation of the bowels.

The dull sound emitted on percussion of the lower and left side of the abdomen led to the conclusion that a large quantity of fæces had accumulated in the colon, and that the intestines were there glued into a mass. There was nothing in the history of the case which would have led the medical attendants to suspect a collection of fluid in a cyst—or at least, in a walled up cavity. There seems to be little doubt that, at some former period, there had been peritoneal inflammation, with some effusion—that the effused fluid had been circumvallated (if the term is allowable) by parietes of coagulable lymph—that chronic inflammation continued in this species of sac—that gradual increase of the fluid took place, by secretion from the parietes of the sac—that some degree of chronic inflammation extended to the peritoneal surface of the intestines generally—and finally, that acute peritonitis supervened and destroyed life.

---

### PARACENTESIS THORACIS IN HYDROTHORAX.

The narrator of the following case very properly remarks that hydrothorax is not invariably the consequence of organic disease of the heart or lungs, and consequently that the discharge of the fluid by paracentesis may sometimes be proper, notwithstanding the sweeping assertion of Mr. Lawrence to the contrary.

*Case.* W. Roberts, aged 40, of an athletic constitution, was admitted into the Lowestoft dispensary, in August 1834. He described an attack of apparent pleuritis in the right side, which happened nine months previously. It was neglected for some days, when bleeding and blistering relieved the pain, but left dyspnœa, which became gradually more and more distressing. There was now a noise on succussion of the right side—no respiratory murmur—dull sound on percussion—evident enlargement of that side. The general health seemed good. A puncture was made between the sixth and seventh ribs, and four pints of sero-purulent fluid evacuated. There was an immediate return of bronchial respiration in that side—the dyspnœa was mitigated, and the wound healed in a few days. The man returned to his business of sail-making, and enjoyed many months of uninterrupted good health. Three months ago he began to experience a return of the dyspnœa, which gradually increased to an alarming degree, aggravated by habits of intemperance. He was re-admitted on the 29th August, 1835, with all the former symptoms, except the fluctuation. The operation was repeated, and nine pints and a half of fluid (same quality as before) were discharged. The breathing was relieved, the wound soon healed, and again the man was discharged, in apparent good health.

We should be inclined to keep the wound open with a tent, so that the fluid might be drained off as it was secreted from the diseased surface, and thus give an opportunity for that side of the chest to contract, and the two surfaces to agglutinate.

---

The Proteian Malady—(Irritation simulating Inflammation.)

This indefinite term has been applied to dyspepsy, hysteria, and to various anomalous forms of neuralgia, resulting from the application of malaria.

Mr. Torbet, of Paisley, has recently published, in our respected northern contemporary, two cases and some observations, which may fairly come under the head allotted to this article. We shall briefly abstract one of the cases.

*Case.* Miss ——, aged 15, of precocious growth, experienced, in the Summer of 1831, an attack of cough and hoarseness, which caused some alarm, but yielded to treatment. In October of the same year, she had a severe attack of typhus, accompanied by bad cough. This, again, was followed by an inflammatory affection of the hip-joint. In February, 1832, palpitation of the heart, with cough, dyspnœa, and quick pulse, was the grievance. The treatment was depletion, rest, and low diet. This plan relieved, but did not remove the symptoms. In October of same year, the family came to Paisley, and the young lady became Mr. Torbet's patient. She looked well, and was loth to acknowledge herself to be an invalid; yet her breathing was quick and short, pulse rapid, and cough troublesome. Various remedies were employed, including digitalis, leeches, and blisters, with little impression on the cough. There was no expectoration. It had a "harsh, ringing, whizzing sound," attended with palpitation, and sense of globus hystericus. From 40 to 50 ounces of blood were taken from the arm at four bleedings, and within six days. No faintness occurred—the blood was neither buffed nor cupped. Temporary relief was obtained by each venesection. On the 5th November, a violent fit of retching occurred. This was attributed to large doses of opium which she had been taking. The cough persisted, with little respite, day and night, being hollow, barking, and so violent as to threaten the bursting of a vessel. Still she did not emaciate, the eye was bright, the tongue clean, and the bowels open. Dr. B. of Glasgow, was called into consultation, and considered the case to be disease of the heart. [We find no mention of auscultation, either by Mr. Torbet or Dr. B.] An open blister was ordered, and two grains of conium thrice a day. In January, 1833, the report was, that the distressing cough has now nearly subsided—the pain and palpitation had diminished. *Per contra,* however, she now was affected with severe headaches, ending in retchings, occurring every second day. Hydrocyanic acid was then substituted for the conium, and augmented till she took five drops three times a day. She also took two grains of opium every night, and a dose of quinine thrice a day. Oxide of bismuth was substituted for the quinine; but with little advantage. At the end of February, the cough had disappeared, together with pain and palpitation, though she could not lie on the left side. The headache and retching continued with great obstinacy. Iodine and a strong chalybeate water were employed. Toothache was now added to the list, or rather swallowed up the other symptoms. Still it ended in retching. Five or six teeth were extracted, with temporary alleviation. They were all more or less decayed. Suspicion now fell on the spinal marrow. The back was carefully examined, and was found generally tender and weak, with a tendency to twist in the lumbar region. She was laid in the horizontal posture on a firm mattrass. Leeches, cupping, and a caustic issue. Large doses of the black drop were given. During three months, the symptoms frequently changed their form and seat—but it was only the substitution of one set or series for another. In the beginning of May, the cough was almost incessant, and very violent, with great aggravation of the pain in the back and chest. The black drop gave very temporary relief. In despair, Mr. T. opened a vein, abstracted some blood, and applied a circular blister round the neck. Temporary relief was the result. A statement of the case was submitted to a high medical authority in Edinburgh (Dr. Abercrombie), who "felt at a loss what to advise." He suggested, however, a course of tonics and purgatives—failing which, he advised stramonium, colchicum, and then nitrate of silver. Sponging, frictions, blisters, and gradual exposure to the open air, were also recommended. The day after this advice was received, hæmoptysis occurred. The usual means were employed, and, on the 26th June, diarrhœa took its turn, but did not last long. The hæmoptysis became rather alarming. On the 14th July, the stramonium was commenced, but seemed to have little effect. The hæmoptysis continued, and increased. There were now evident inflammatory symptoms, and blood was taken pretty freely. It was cupped and buffed. Examination by the stethoscope was now first mentioned; but Mr. T. states that he had used the instrument several times before, without being able to draw satisfactory conclusions. The hæmoptysis continued, with more or less violence, till the middle of August, when violent cramps on the abdomen added to the misery of this unhappy girl. They were accompanied by severe vomiting. Mercury was now combined with the stramonium. In about a week, the amazing quantity of fæces daily passed astonished the medical attendants. "Often there was half a chamber-potfull of solid fæces, loose and ill-digested, yellow, granular, and very offensive." It was difficult to reconcile these large egesta, with the small quantity of ingesta. But we find it impossible and unprofitable to follow the narrative of this extraordinary case. Suffice it to say, that there is scarcely a disease in Cullen or Good's Nosology, which had not its type or representative in the person of this poor young lady—catalepsy not excluded. CHANGE of AIR appears at length to have been the sovereign remedy, though she is yet far from well. A removal from the miasmal grounds about Paisley, to the bracing breezes of Stirlingshire, effected wonders—and this long-afflicted damsel may yet regain her health.

The following observations are worthy of notice.

"*Neuralgia*, a term which I use as synonimous with spinal, ganglionic, or cerebral *irritation*, may attack any part of the nervous system, from the minute fibrils spread on the organs of sense, to the great nervous centres. The two cases subjoined, I regard as examples of the phenomena occurring when the centres of the system are attacked. In attempting a general outline of such diseases, it is vain to look for any thing like unvarying regularity of symptoms. We know nothing of the exact morbid condition giving rise to the symptoms. It may be of various kinds; and, besides, in so delicate and complex a tissue as the nervous system, we may easily conceive, the many modifying and controlling circumstances, that cannot fail to occur, in each individual case, when that system becomes the subject of morbid irritation, and thus vary the external phenomena. But though systematic exactness be out of the question, it is still possible to give something like a characteristic outline.

Of the diseases in question females frequently before, sometimes after, puberty, are the most usual victims. The individuals are of what is called irritable constitution—a peculiarity, moreover, in some instances, distinctly hereditary, and connected more or less with strumous diathesis.

The morbid train of neuralgic symptoms is most usually set in operation either by a serious inflammatory or febrile disease, by excessive fatigue, or by injury, as from a heavy lift or severe fall. The severity and variety of diseases to which the individuals appear to be liable might almost characterize the affection. You are astonished to hear of their violent illnesses, the powerful remedies that are applied, and the rapidity with which they shake off these attacks. In severity, their diseases resemble those of no other person. They are paralytic, blind, speechless, without food, and without sleep for weeks or months, and yet they do not *die*.

Is the *head* the organ affected? They have headach, of which no words can convey an idea, ending perhaps in insensibility, or there is a heavy weight pressing down their eyelids, a tight bandage compresses, or hammers beat within their head, bells ring in their ears, vertigo and sickness overpower them, noise and light disturb them.

Is the *chest* affected? A sepulchral cough, each fit of which seems to threaten existence, over which medical art seems to possess no control, harasses from morning till night, and night till morning. There is excruciating pain in the chest, especially during the cough, which is perfectly devoid of expectoration. There is a feeling of swelling and choking at the under part of the neck, tremendous palpitation of the heart; the pulse is quick and agitated, but varying greatly with the severity of the cough; and the heart's pulsations seem to occupy the whole left side of the chest.

Is the *digestive apparatus* the seat of disease? Then ensue vomiting without apparent cause; incessant retching for hours, occurring perhaps every day for weeks, without any thing being vomited; no appetite; unquenchable thirst; occasional severe pain in the belly, or muscular cramps; and costiveness alternating with diarrhœa—the stools unaccountably copious.

Are the *organs of voluntary motion* the parts attacked? There are then cramps, prickling or numbness, sometimes severe spasms, liker tetanus or hydrophobia than any thing else, ending possibly in catalepsy, or paralysis of one or more limbs ensues.

Such is an epitome of the symptoms of the more violent cases; but almost endless variety must of necessity occur, according as the several nervous centres are affected and their affections modidified, by individual circumstances. It is precisely from this variety and anomaly of appearances, that practical men are apt to be misled. The most marked symptoms are apparently so distant from, and unconnected with, nervous irritation, and are moreover so violent and similar in their character to pure inflammatory diseases, that there is great risk of our chief curative efforts being directed to the appendages and coincidents, instead of attacking the disease itself."

This is all very well. But how are we to get at the disease itself? How are we to get at its seat, if in the ganglionic system of nerves? That system is, as it were, withdrawn from our reach. In attacking the spine, we assail the system of voluntary nerves more than we do the involuntary; and, therefore, when we apply issues to the back, we cannot be certain that their beneficial effects are produced through the medium of the ganglionic nerves.

---

### EASTERN PROVINCIAL MEDICAL AND SURGICAL ASSOCIATION.

We rejoice to see that another provincial medical society is formed, for the purpose of uniting the practitioners of Cambridge, Essex, Huntingdon, Lincoln, Norfolk, Suffolk, and other eastern counties, in one common object—the promotion of medical science, the respectability and harmony of the profession, and the publication of transactions. It is nearly on the same plan as the Western Provincial Association, and promises to be of great utility. It appears to have been set on foot through the instrumentality of that zealous and talented member of our profession, Mr. Crosse, of Norwich. The first meeting is to take place at Ipswich, early in June next, when the society will be fully organized, and actual proceedings commence. Mr. Crosse made an eloquent speech at Bury St. Edmonds, in September last, when upwards of 70 medical gentlemen were assembled, in consequence of a public requisition. We have no doubt that this association will rival its parent institution in the West, and that great benefit will accrue to science and humanity from the conjoint labours of so many individuals.

---

### PUNCTURE OF THE BLADDER ABOVE THE PUBES.

The following interesting case is published by Dr. Geddes, in the first number of his "NORTH AMERICAN ARCHIVES."

*Case.* "Thomas Oliphant, aged 28, by trade a carpenter, was admitted into the Baltimore Infirmary, on the 24th of August, 1832, laboring under a total suppression of urine, which had existed about forty-eight hours. His bladder was distended into a large round tumour, which projected considerably above the symphysis pubis; his whole abdomen was extremely tender to the touch; his pulse frequent and small, and his skin covered with a cold clammy sweat. His whole aspect indicated extreme suffering, and his condition excited much apprehension for his safety.

The account furnished by himself of the origin of his disease was, that some weeks before, whilst engaged in his occupation, he slipt and fell from a height, astride of the edge of a plank, which struck him across the perineum, immediately in front of the anus, bruising the parts violently, and occasioning a temporary stoppage of his urine. From this latter he partially recovered in a few days, in proportion as the inflammation subsided; but although able to relieve his bladder, the urine did not flow freely, and he frequently experienced considerable difficulty in passing it. After the lapse of some time this difficulty increased, until it finally amounted to a complete suppression, to obtain relief from which, he entered the Infirmary in the condition already described, unsuccessful attempts having been previously made by his medical attendant to pass the catheter.

On passing an instrument down the urethra, I found the obstacle to be situated immediately behind the bulb, and in the membranous portion of the canal, where the parts seemed to be much indurated, and presented a solid point of resistance both to the instrument and the finger. This induration seemed to reach backwards to the extent of several lines. Various attempts were made to overcome the obstacle, but all of them were fruitless. The patient was then put in a warm bath and bled ad deliquium, after which the attempts to pass the catheter were renewed, but with no better success. Laudanum was administered in large doses both by the mouth and anus, but still the obstacle could not be surmounted. The patient was then left for a couple of hours, with the direction to keep the whole abdomen covered with warm anodyne fomentations, and to have a large anodyne enema. On my return, I renewed the efforts to introduce the catheter; but the obstacle was still as formidable and unyielding as before. The sufferings of the patient being extreme, and his danger hourly increasing, I determined to resort immediately to the puncture of the bladder; and as there was no trocar at hand by which the puncture could be made in perineo, I proceeded to perform the operation above the pubis, in the following manner:—A sharp pointed bistoury was thrust directly backwards into the bladder immediately above the symphysis pubis and upon the lower part of the linea alba. A female catheter was then conducted along the blade, through the opening, while the knife was withdrawn simultaneously. The urine was drawn off through the catheter, which was fixed in its situation by means of tapes attached to its eyes and passed round the body. The relief was immediate, and continued, the plug of the catheter being from time to time removed, to draw off the urine whenever it accumulated.

From this time the patient was left to quietude, and such treatment as was calculated to subdue the irritation excited by the disease and operation, until the third day, when every thing being favorable, renewed attempts were made to overcome the obstacle in the urethra. The ordinary methods having been perseveringly tried without effect, I determined to resort to an operation in perineo for the re-establishment of that passage.

The patient being placed in a convenient position, with his thighs flexed upon the pelvis, a silver catheter was passed down to the obstacle in the urethra, where it was held firmly by an assistant, in such a manner as to make its extremity project a little in the perineum, in order that it might serve as a guide for the knife. An incision was then commenced upon the raphe of the perineum, immediately behind the bulb of the urethra, and conveyed backwards in the direction of that canal, as near as possible to the verge of the anus. The skin and integuments being thus divided, I dissected down cautiously upon the end of the catheter, to reach which, it was found necessary to divide a part of the bulb. This point gained, and the direction of the natural course of the passage thus ascertained, the dissection was cautiously continued backwards, through the indurated tract of the obliterated membranous portion of the urethra, with the object of reaching the open portion of the passage posterior to the obstacle. This was not accomplished until the anterior extremity of the prostate gland was reached, where, after some little impediment, the point of the catheter was insinuated into the canal and conveyed immediately into the bladder.

There was no vessel divided requiring a ligature, and after sponging the parts dry, the patient was put to bed, with his thighs placed close together, and the catheter properly fixed in his bladder by means of tapes and a bandage. On the second day after the operation, the silver catheter was withdrawn, and an elastic one introduced without difficulty. Only a small quantity of urine flowed through the wound, and the only subsequent attention requisite was, to change the instrument from time to time, whenever it became foul or obstructed.

The natural course of the urine being thus established, the female catheter which had been introduced above the pubis, was removed, and that aperture closed in two or three days. The wound in the perineum healed kindly, and the individual, after remaining in the house some time to ensure the permanent permability of the urethra, was discharged on the 28th of October, perfectly cured."

---

On Diseases of the Stomach; their Sympathies and Complication. By Mr. Langston Parker, of Birmingham.

This paper is published in the September number of the Dublin Journal, and evinces accurate observation and clear views of a class of diseases very complicated and obscure in their nature and etiology. The class to which Mr. Parker particularly directs his attention in this paper, is the "*morbid sensibility*" of Dr. Johnson—the "*nevroses des visceres*" of the French pathologists. Mr. P. justly remarks that the separate textures of the stomach may be the seats, separate seats of the causes of impaired digestion, in addition to which "the vascular and nervous systems entering into the composition of the organ may be in this, as in all other parts, separately the seats of disease, so as to render the affection of one order of parts the predominant feature of the disease." Mr. P. then proceeds to illustrate the following position, by a series of cases.

"Morbid sensibility of the stomach and intestines, gastralgia or enteralgia, are terms made use of to designate a state of disease in which the mucous surfaces of the alimentary canal are painfully sensible to impressions which in a healthy condition they do not perceive; these impressions, by the exaltation of nervous sensibility which accompanies them, materially deranging the functions of those organs in which they are seated, and by their reaction upon the economy at large, producing sympathetic diseases of great variety and importance.

Morbid sensibility may occur either as a primary affection or succeed to attacks of an acute inflammatory character, whence it is apt to be mistaken for a recurrence of the inflammatory disease."

*Case* 1. A delicate lady, aged 23, had a painful affection of the stomach after taking food of any kind, accompanied by acid eructations, and slight constipation. It had been of only three weeks' duration, and yet induced considerable emaciation. She had profuse night-sweats, with cough, and great depression of spirits. The chest exhibited no evidence of disease, when examined by the stethoscope; but the epigastrium was slightly sensible to pressure. She was put on farinaceous diet, and took a preparation of rhubarb, aloes, and morphia, with hydrocyanic acid. She recovered in a fortnight, and was able to eat any kind of food.

*Case* 2. A lady consulted the author for a painful affection of the stomach, whose features were precisely the same as those detailed above. She had had an acute inflammation of the stomach 14 months previously, preceded by symptoms similar to the present ones. The same diet and medicine as were given in the preceding case, and in ten or twelve days she was able to take food without inconvenience.

These two cases are adduced as "the simplest forms of morbid sensibility;" but from this we dissent. There are many grades and shades of morbid sensitiveness in the gastric nerves below the point of actual pain, and where a host of wretched sensations are experienced, of a mental rather than corporeal character, of which neither physician nor patient can give any intelligible description. In fact, no medical man becomes acquainted with the subject till he has experienced the miseries of indigestion! Mr. Parker has not fathomed half the depths of this Proteian malady.

Mr. Parker traces sympathetic affections of the chest much more frequently to disorder of the stomach than we have been able to do. At the same time we do not deny that the one may be a consequence or sequence of the other.

*Case* 3. A delicate man, aged 32 years, had been subject for six months to pain in the stomach on taking food, which is occasionally rejected by vomiting. His state on the 3d July, 1834, was as follows:—countenance anxious, skin cold, breathing hurried, constant cough, expectoration of mucus. *The chest was examined by percussion and auscultation, and found to be apparently healthy.* To this we demur. It is impossible that there can be constant cough with expectoration of frothy mucus, independent of a catarrh of the mucous membrane of the air-tubes. But to proceed:—there was great tenderness at the epigastrium, confined to a small surface. The food was constantly rejected, with great pain—tongue coated—papillæ red and enlarged—pulse hurried and bounding—urine high-coloured and scanty. Six leeches to the epigastrium, and afterwards a blister. Rhubarb, acetate of morphia, and hydrocyanic acid were prescribed. Farinaceous diet. On the 7th, it was found that the food had remained on the stomach without pain. The cough was gone, and the pulse was reduced. He was well by the 15th of the same month.

*Case* 4. This was a complication of morbid sensibility with acute inflammation.

" A lady, some months since, three days after parturition was carried out in her chemise, and laid upon the grass, in consequence of her house taking fire. Since then she has been constantly troubled with acute pain after taking food, which is constantly rejected by vomiting.

Present state, five months after the exposure:—great anxiety of countenance; extreme weakness; constant pain in the stomach, increased by pressure to agony; cold skin; small quick pulse; relaxed bowels; stools dark and offensive; incessant cough, with abundant frothy expectoration. (Chest examined by auscultation and percussion, apparently healthy.) Under a treatment composed of local depletion, blisters, the acetate of morphia, and hydrocyanic acid, this patient speedily recovered."

After such an exposure as the above, we can see no reason why the pulmonary affection should not be engendered by cold, as well as the gastric.

After detailing some other cases, Mr. Parker goes on to remark on sympathetic affections of the brain, heart, &c. consequent on irritation of the gastric nerves. He then gives a case of—

" *Gastrodynia with Hallucinations, and Disposition to Suicide.*"

A gentleman had uneasy sensations in the stomach, increased after meals, with occasional vomiting, distention, acid eructations, and some epigastric tenderness. Leeches and blisters relieved him for a time; but Mr. P. was afterwards summoned to the patient, who was found to be labouring under great mental uneasiness, pain in the head, heat about the temples and forehead, acute pain in the epigastrium, frequent vomiting of acid fluids, pinched and anxious countenance, cold skin, frequent pulse, tongue coated, and red at the tip. The paroxysms of pain after eating are now terrible, till the stomach has rejected the food. His nights are restless and his dreams distressing. When awake he is harassed by hallucinations of various kinds, the chief of which is a blackbird flying against his head with great force, and producing, as he thinks, the headache. In the day time he has an almost irresistible propensity to suicide. He was confined to farinaceous food, had leeches to the temples, and took infusion of cascarilla, magnesia, tincture of aloes, and liq. opii sed. every four hours. There was no hallucination in the night after the leeches, and the suicidal propensity was diminished. A day or two afterwards, he injudiciously dined on animal food, which was followed by an exasperation of all the symptoms. A blister was applied to the epigastrium and the same remedies continued. He gradually improved; but had a violent relapse after imprudence in diet, from which, however, he recovered. We agree with the author that " the hallucinations were strictly dependent on the morbid sensibility of the stomach."

" The importance of restricting the patient to a pure farinaceous and unstimulating diet, during such affections as the present, is well illustrated by this case. As long as the food is mixed or stimulating, no medicines will afford relief; and if a plan of diet of this kind were established, or an irritating medical treatment employed, the patient would inevitably either become deranged or commit suicide."

Mr. P. proceeds to state some cases of epigastric pulsation, which he attributed to morbid sensibility of the stomach.

*Case* 5. A lady, aged 40, had menstruated irregularly for three years past. Her food occasioned great pain, accompanied by nausea, and sense of great distention. There was also a sense of rolling in the stomach, with intense acidity. Carbonate of magnesia relieved these symptoms, with the aid of a blister. Great despondency succeeded, with indescribable sensations in the stomach—nausea, and sometimes vomiting. There was a strong pulsation in the epigastrium, evident to the eye, and accompanied by dull pain increased by pressure. Skin hot and dry. Every kind of medicine increased the pain.

Leeches were applied every other day for six days, and then blisters. The more urgent symptoms yielded to this treatment; but the mental depression, pyrosis, and occasional vomiting continued. "Hard farinaceous food" was the only aliment she could bear.

Another case is shortly stated in which pain after food was complained of, with occasional vomiting and epigastric tenderness. There was constant and strong pulsation in the epigastrium, visible to the eye. These symptoms disappeared after a few applications of leeches and blisters.

In a succeeding case a lady was attacked with typhus fever, and, in the course of convalescence, the stomach became so irritable and painful on taking food, that it was impossible to take sustenance. "The mental uneasiness accompanying this state was distressing in the extreme." She rolled from one side of the bed to another, constantly complaining of her stomach, for some weeks. Yet the tongue was clean, the pulse quiet, skin cool, and no increase of pain in the epigastrium on pressure. Opiates, alkalies, and tonics aggravated the uneasiness. Carbonate of iron was exhibited, but "its effects were frightful." Medicines were omitted altogether—the patient was removed into the country—"the mildest food, in very small quantity, was given, and, after a long time, she recovered." We have seen many cases of this kind, and they have been truly denominated "MORBID SENSIBILITY of the stomach and bowels." We are unable to pursue the cases detailed by our author; but shall make room for his general conclusions.

"*Recapitulation.*—It will be at once seen, from a perusal of the sixteen cases I have detailed, that although they were analogous in one great point, that of disease of the stomach, they were in most others very variable. We find the simplest forms of disease commencing in mere uneasiness, hardly amounting to pain after taking food, and thence progressing into violent gastrodynia, to sympathetic affections of the organs of respiration, in three instances terminating in acute inflammatory diseases. In seven cases, the organs of respiration were the seat of either nervous or vascular lesions, consequent upon the stomach disease. In four cases, mental disturbance was present, in one instance producing hallucinations, and disposition to suicide, and a degree of despondency almost approaching the insane state in the other three.

1. *Complication with Gastritis.*—In two cases (III. and IV.) the disease, (i. e. the morbid sensibility, or irritability of the stomach or gastrodynia,) was evidently complicated with inflammation, and in two others (V. and VI.) it succeeded to a lesion of this character. The latter circumstance at once stamps it as an affection of a distinct character from inflammation, although it is frequently complicated with, and inflammation is frequently produced by it. Where morbid sensibility has been present many months, it sometimes becomes complicated by sub-acute or chronic gastritis, and in these instances much more circumspection is requisite in ascertaining clearly the history of the disease, and in applying the remedial agents, than in any other form of stomach complaint. Here a combination of treatment is required; an antiphlogistic, and a sedative one, and while a moderate local depletion is absolutely necessary on the one hand, the internal remedies should be of the character made use of in the details of the preceding cases. Whilst we are leeching the epigastrium, the internal treatment cannot be too mild, or unirritating, but must be varied, of course, to suit the particular circumstances of the patient.

2. *Lesions of the Respiration.*—In seven out of the sixteen cases related, lesions of this character were observed; in the more simple forms, we had present merely spasmodic cough, and hurried breathing; in others, frothy expectoration; in one, bloody expectoration; in two, pneumonia. In most, no physical sign of disease of the respiratory organs was present, in two, slight râles accompanied the cough, and in two others the physical signs of pneumonia were unequivocally present. In all, the lesions of the respiratory organs disappeared, as the disease of the stomach was mitigated or cured.

3. *Lesions of the intellectual Faculties, and of the Senses.*—These lesions were observed in four of the cases I have detailed, and chiefly consisted in impaired judgment, and excessive despondency; in one, the disposition to suicide was almost irresistible; this case was also remarkable for hallucinations of the sense of vision. I have, in the remarks on this and other cases, alluded to instances in which the same lesions of the intellectual faculties have terminated in actual and confirmed insanity. All these affections of the mind were in strict relation to the degree of the morbid sensibility of the stomach, and were aggravated, and subsided with it.

4. *Lesions of the Circulation.*—These chiefly relate to the pulsations in the epigastrium, observed in three of the cases. I do not know to what exact pathologic condition to refer these pulsations. They were all accompanied by local determination of blood, and heat in the epigastrium, but the symptoms which accompanied them certainly did not resemble pure gastritis. My own conjecture is, that they are owing to irritation of the great nervous ganglia, and plexuses of the epigastrium, lying behind the pyloric portion of the stomach, and ramifying upon the vessels of the cœliac axis, and arterial system of the abdomen generally; for in one instance the pulsations might be followed along the course of the aorta to some distance. Whatever might have been the primary nature of these irritations, they were evidently accompanied by increased local vasculai action, and were relieved in all the examples by depletion; in all they ceased on the subsidence of the stomach disease."

These cases are deserving the attention of the profession. We have seen such deplorable effects from the routine practice of tonics and drastic purgations by some—and inordinate depletion by others, that we cannot too strongly urge the necessity of cautious discrimination on the part of the practitioner. Mr. Parker certainly has not investigated with sufficient care the etiology of the complaint. He has not traced these gastric affections, so closely as he ought to have done, to their primary sources. If he had done so, he would have found *moral* causes playing a deeper part in their etiology. He has adopted the designation which we were the first to apply to this extensive and afflicting malady; and for this we thank him.

---

### Pulmonary Apoplexy.

From a volume of hospital cases lately published by Dr. Aldis, we select the following.

" Mary G., æt. 33, admitted *July 17th,* 1833.—Has had seven children, the last born five years ago; the swelling in her legs began three weeks ago. In the winter she felt a severe beating of the heart when she went up a hill or up stairs, and about two months back, a lump began to grow at the pit of the stomach, which affected her breathing. Great action of the heart; dyspnœa; cannot lie down in bed. Pulse intermittent and very irregular, 65, very weak; bowels not open; urine with a reddish brown sediment; catamenia irregular.

A large hard protruding tumor, occupying both hypochondriac and epigastric regions, descending to the right lumbar and umbilical region. Two nights ago a purple eruption broke out upon them.

*Note.*—I examined the heart with the stethoscope, and found very strong impulse of the right ventricle; there was no bruit, and no increased sound. She suffered from great dyspnœa, and hæmoptysis. On September 3rd she made use of a sentence similar to one employed in Aretæus to express the redundant secretion from the skin. She complained the perspiration was so great last night that it " seemed as if she had been dragged through a river.*"

*9th.*—Died at 10 P. M.—*Sectio.*—1 P. M.

---

*Thorax.*—There was found in the left side of the chest about a pint of bloody serum, and also a small quantity in the right; the left lung adhered firmly to the ribs, and had imbedded in it a hard rugged substance, of the size of a large marble. The lungs were, in many parts, hepatised, of a grey colour, in circumscribed masses and portions so gorged with blood, that it resembled black currant jelly. (" Apoplexy of Lung.") The heart was very large, and firmly adhering, at its apex, to the pericardium. Both ventricles were much dilated, and the parietes of the right somewhat thickened. *The mitral valves were much thickened with a deposit of ossific matter near their opening.* The appendix of the left auricle contained a quantity of hard laminated coagulum, which had nearly lost its colouring matter.

*Abdomen.*—The liver was very much enlarged and of an unhealthy structure. The os uteri was very much congested, and the substance of the womb, both in cervix and fundus, much enlarged and hardened, of an uddery consistence.* Its cavity was very small. There were many dark superficial ulcers in the upper part of the vagina.

*Remarks.*—A paper was read at the Royal College of Physicians, London, in 1829, showing the dependence of pulmonary apoplexy on disease of the mitral valves. Three cases, which occurred at St. George's Hospital, were brought forward to support the author's views. Hæmorrhage from the lungs is very common in apoplexy of the lungs; the blood is propelled from the right ventricle of the heart through the pulmonary artery, it is then brought to the left auricle by the four pulmonary veins, and from thence it enters the left ventricle. The mitral valve, when thickened with warty vegetations, &c., is incapable of performing its office, the blood regurgitates, hæmorrhagic indurations are formed in the lungs, and expectoration of blood is the consequence." 114.

* Not schirrous, because not gristly, a state of the womb, I believe, not described in books.

ANÆMIA.

Dr. Geddes has published some cases of Anæmia, in the first number of his Archives, from which we extract the following one.

" Charles Winn, seaman, aged 18, was admitted into the Baltimore Infirmary, July 30th, 1834. He was born in a malarious district of North Carolina, and from his earliest youth has been annually subject to intermittent fever, until two years previous to his admission, during which time he has led a seafaring life.

The whole body at the present time is exsanguined, more or less mottled, and of a pale waxen hue. His cheeks and ankles are œdematous; the abdomen tumid; adnata of the eye of a pearly lustre; lips, gums and tongue blanched, and the latter pointed; muscles flaccid; body emaciated; digestive powers partially impaired; respiration much disturbed, the least exercise occasioning hurried and oppressed breathing, and if continued producing speedy exhaustion. The heart palpitates violently even when lying at rest, and there is tumultuous throbbing of the carotids and other large vessels about the neck. The pulse is also frequent, and presents a peculiar *shattered thrill.* The liver and spleen are perhaps enlarged, but the effusion of water into the abdomen renders it difficult to decide by exploration. Massa. Pil. Hydrarg. gr. iij. Pulv. Soc. Aloes, gr. j. Pulv. Ipecac. gr. ss. every night. Carb. Ferri, gr. x. three times a day. Generous diet with porter.

*Aug.* 10. The improvement of his general health has been steadily progressive. Tongue and lips slightly reddened—abdomen less tumid. The œdema of the cheeks and ankles has lessened, digestive function invigorated, and the evacuations more healthy.

*Aug.* 25. Still improving. Tinct. ferri muriatis substituted for the carbonate of iron. Alterative pills to be continued.

*Sept.* 11. All the prominent symptoms of the disease have yielded, in a great measure, to the treatment. Tongue and lips more colored; the tumefaction of the abdomen has entirely subsided, as has the œdema of the cheeks and ankles. The palpitations of the heart, and the excessive throbbing of the vessels are no longer perceptible. His pulse is regular and full, and he is able to bear considerable exercise without fatigue." 41.

---

### Intestinal Convulsions.

A fatal case of this kind is related by Dr. M'Leod, of Bridlington, which is worthy of notice. A child, three years of age, ate a large quantity of fruit during a very hot day. In the evening Dr. M. found her labouring under pain and tumefaction of the abdomen, with pallid countenance, quick pulse, white tongue, urgent thirst, knees drawn up to the abdomen. Convulsions came on, and followed each other in quick succession. The warm bath was used, and calomel and rhubarb were given; but without any good effect, and the child expired in a strong convulsion early next morning.

On dissection, there was found a quantity of half-digested unripe fruit and peas, in the stomach, which was also much distended with air. There was no appearance of inflammation. The whole of the intestines were remarkably distended with fœtid gas. They were free from inflammation. The cystic bile was "dark-coloured and unhealthy." There were no morbid appearances in the brain or other viscera.*

Dr. M'Leod's ratio symptomatum, or pathology of the flatulency, is rather curious. "A healthy flow of bile is unquestionably indispensable to the peristaltic motion of the intestines; and, on the other hand, a *vitiated secretion of that fluid must retard their vermicular motion,* and tend to detain acrid alimentary substances in the duodenum, &c." "The accumulated exposure to animal heat consequently occasions the acetous fermentation, the disengagement of gas, excessive *pressure* on the extremities of the superior mesenteric, and branches of the solar plexus of nerves which enter the viscera of the abdomen, and sympathetic affections of the limbs and other parts of the body, producing convulsions, and such depression of all the powers of the system, as, unsubdued, must terminate fatally."

From some attention to the subject we can assure Dr. M'Leod that a healthy flow of bile is not *indispensable* to the peristaltic motion of the intestines, since we often see the bowels perfectly regular in the most intense jaundice. On the other hand, so far is vitiated bile from causing torpor of the bowels, that it is one of the most efficient causes of bowel complaints. We think Dr. M'Leod has refined very much respecting the cause of death in the above case—namely *pressure* on the splanchnic nerves, in consequence of the flatulence. To us the cause of death appears to have been the irritation of unripe and undigested fruit in the *stomach,* inducing convulsions by the well-known sympathy between the *brain* and that organ. The child, in fact, was poisoned, and presented most of the phenomena of poisoning, except vomiting. And here we would venture to suggest the propriety of exhibiting an emetic under such circumstances as the foregoing. We cannot but think that the dislodgement of the irritating matters from the stomach, in the above case, might have afforded considerable chance of recovery.

---

### Provincial Medical Education.

We are gratified to learn that the system of provincial medical education proceeds in an encouraging manner, especially in the larger towns, as Manchester, Birmingham, Liverpool, Bristol, &c. None but an ignoramus or a thorough-paced monopolist will deny that excellent clinical instruction may be afforded in large provincial hospitals. In the present state of things, indeed, we do not think that a surgeon's education ought to be considered complete,

---

* Lancet, 19th Sept. 1835.

without some metropolitan study and observation, more especially as regards anatomy and operative surgery. But we are clearly of opinion, that the hospitals and infirmaries of the large towns throughout the Empire will very soon be so organized, as to afford all but the full accomplishment of a complete medical education. The following extracts from a very sensible and well-written introductory discourse, delivered by Dr. Bardsley, at the commencement of the tenth session of the Manchester School of Medicine and Surgery, 1835, will bear us out in these remarks.

"This vast and wealthy commercial town, inferior to none in the enterprise, industry, and mechanical ingenuity of its inhabitants, embraces, according to the census returned to Parliament in 1831, together with Salford and the adjoining townships, a population of upwards of 270,000; and we are all aware of the prodigious addition made to that number during the last four years. Within the circumference of twelve miles are also situated the flourishing towns of Stockport, Bolton, Oldham, Rochdale, Ashton-under-Lyne, Bury, &c. each containing many thousand inhabitants, and contributing its share of disease to our different charitable institutions. It must also be recollected, that this active population is constantly engaged in manufactures, and therefore liable to those frequent severe casualties, of which machinery is almost unavoidably the fruitful source. Many accidents, too, attended with danger to life or limb, almost daily occur amongst that numerous class of persons engaged in building, and in the neighbouring collieries. Here, then, Gentlemen, a field presents itself for the study of medicine and surgery, unequalled in the extent and variety of its soil, requiring for its cultivation many diligent and enterprising husbandmen, and repaying by its practical fertility their labour, humanity, and skill."

"Most of the large towns in the provinces now possess general hospitals and infirmaries, established upon the most liberal and extensive principles. In this respect, Manchester is most favourably circumstanced. Amongst other noble monuments of public beneficence, the Royal Infirmary stands pre-eminent, both as regards its early foundation and the extent of its usefulness. In its general management and internal discipline, it will bear comparison with any similar establishment in the kingdom; and whilst the names of Percival, White, Ferriar, Bardsley Senr., Simmons, Home, Henry, and Roget, are associated with its medical history, no lapse of time can impair its well-earned reputation. When it is stated, that during the last year the total number of patients admitted on the books of the charity was 19,467, including 4058 accidents; that 135 capital operations were performed in it; and that it is capable of accommodating within its walls 200 patients, no further evidence will, I imagine, be required to shew, that it affords students most excellent opportunities of witnessing disease under its diversified forms, of contrasting the results of different methods of practice, and of becoming intimately familiar with the future duties of their profession."

"The injustice of metropolitan monopoly will be manifest, when it is mentioned, that this extensive Institution does not enjoy equal privileges respecting certificates of attendance on the surgical practice, with some of the hospitals in London containing scarcely fifty beds. To two applications made by the Surgical Officers of the Infirmary to the Council of the College of Surgeons in 1830 and 34, to have that institution placed on the same footing with the hospitals in London, Dublin, Edinburgh, Glasgow, and Aberdeen, the following was the answer returned by the Court of Examiners of the College: 'We cannot comply with your request, because sufficient time has not elapsed to enable us to form an equitable judgment as to the education of pupils coming from provincial schools.' The profession will judge rightly of the nature of this decision, in the face of the unsolicited testimony given by another Examining Body before a Committee of the House of Commons—'that no class of pupils is better prepared than those who have been educated solely at Manchester.' The spirited memorial of the Surgeons, just noticed, firmly maintains that, owing to the general diffusion of knowledge and increased capability of appreciating useful establishments, the dignity and respectability of the College cannot be preserved without a recognition of such institutions as are acknowledged by public consent to be fully competent to the essential purposes of professional education."

"The contrast between the present and former situation of a provincial pupil, in entering on his studies in London or some other metropolis, must strike every one who reflects at all upon the subject. His mind may be compared to a soil that has first been duly cultivated for the reception of seeds; which having advanced to the growth of young shoots require only time and further training for their full development. According to the former appointed plan, of two winters' residence in London, the greater part of the pupil's time was consumed in obtaining that elementary knowledge of anatomy, and the other branches of medical science, with which he is now familiar before he leaves the provinces; and thus he is prepared at once to take advantage of the more enlarged field of observation presented to his view, and to collect and arrange new materials for further study and reflection. The remark of the Latin Poet is applicable in his case:—' Dimidium facti, qui cæpit, habet.'—' He has half done, who has made a beginning.'

It is obvious, then, that provincial schools, so far from being calculated to supersede, or interfere with the courses of instruction pursued at the metropolis, on the contrary, directly tend to increase their usefulness."

In the above sentiments we entirely agree; and sincerely hope and expect that the generous rivalry of the provincial with the metropolitan teachers, will greatly increase the general amount of medical education, and, consequently, diminish the sufferings of human nature.

---

### On Scarlet Fever. By Dr. Sandwith.

The author was induced to send an extended paper on scarlatina to the Medico-Chirurgical Society—which Society commended the communication, but declined its publication. It was afterwards inserted in our northern contemporary, and is now re-published, as the first of a series of practical commentaries on some of the most important diseases of children. We shall pay due attention to the forthcoming essays of Dr. Sandwith, knowing him to be an observant and talented practitioner. It cannot be expected, however, that, under the existing circumstances, we can more than very briefly notice the present essay. The following extract is best calculated to exhibit the nature of the pamphlet.

"The epidemic scarlet fever, which it is my task to describe, exhibited the following varieties:—

1. The majority of cases manifested a high degree of fever, with more or less of delirium, and an intense inflammation of the fauces. With considerable irregularity in the development of animal heat, however, in this, as in the severe epidemic described by Dr. Bateman, the period when cold washing promised to be remedial was soon over; but tepid ablutions were applicable throughout. Whether the brain was more or less implicated, the throat almost invariably ulcerated or sloughed, especially when local depletion was inadequately employed, or trusted to alone. In neglected cases of this kind, the extension of the inflammation, from the mucous membrane of the fauces to the excretory duct of the parotid and submaxillary glands, exasperated, doubtless, by the sympathetic irritation dependent on the rapid sloughing of the throat, produced excessive tumefaction of those glands and the surrounding cellular tissue, with consequent mechanical obstruction to the return of blood from the brain. A low fever, with delirium, sometimes slowly, sometimes rapidly, terminated their sufferings. In the latter stages, from the extension of the inflammation to the larynx, this variety was prone to lapse into the fourth, or that of bronchial inflammation. All were not attacked with equal severity, the symptoms being mild in a few instances.

2. In some of the cases gastric irritation, from the extension of the inflammation of the fauces down the œsophagus to the stomach, greatly augmented the danger, by adding to the extent and importance of the inflamed surface of mucous membranes, interrupting the rash, rendering the operation of purgatives more precarious, and exasperating the irritation of the nervous system. Occasionally the whole alimentary canal seemed to participate in the affection of its upper portions. The fatal epidemic of 1793, from Dr. Lettsom's description of it, obviously corresponded closely with this variety. It differed, at the same time, from those described by Drs. Fothergill and Hamilton, because decided affections of the abdominal portions of the alimentary canal were comparatively rare in this epidemic.

3. In several cases active delirium, or coma, set in at once, or within a few days, after a delusively mild appearance of the disease In the former, the excitement ran extravagantly high, the patient singing and raving by turns, until signs of oppression supervened on those of exalted sensibility. In the latter, there was involuntary evacuation of the fæces within a few hours of the attack. The eyes were ferrety in both, but muddier in the latter; in which, also, the skin was cooler, and the rash duskier. In one example, which partook of the characters of both these forms of cerebral disorder, there was extreme coldness of the extremities, with blueness of the fingers and lips; and petechiæ mingled with the eruption. Life was extinguished, in these cases, within two, three, or, at the most, four days after the development of the cerebral symptoms. The throat was sometimes severely inflamed; at other times the transition of the inflammation to the arachnoid membrane was almost complete. *Subsultus tendinum* occasionally appeared very early.

4. In a still greater number of cases than those of the third variety, a marked bronchial inflammation, with consequent sensorial oppression, sometimes extinguished life with equal rapidity; in other instances, in which it came on later by an extension of the *cynanche tonsillaris* to the trachea, death ensued in a week or ten days. In this form of the disease, the rash was invariably interrupted, faded, assumed a copperish hue, or wholly disappeared. The pulse was quick and feeble; the skin, for the most part, cool; the tongue dry and glazed; and the whole system rapidly laid prostrate.

I also witnessed some clear examples of the disease, which arranged themselves under none of these varieties, but corresponded with the irregular congestive form described by Dr. Armstrong. The most striking features were, the cold stage, with repetitions of it so intense, as to resemble approaching collapse; occasional paroxysms of febrile heat, observing no periodical exacerbations; excessive irritation of the nervous system; the rash faintly emerging and disappearing, but never establishing itself; black and offensive stools; the throat but slightly inflamed; the pulse varying most remarkably in frequency; and the tongue furred with red papillæ, but always moist.

The following were among the most prominent sequelæ of the disease: General dropsy occurred, in a few instances, from exposure to cold during convalescence, but proved manageable. In one instance, not resulting from cold, but obviously dependent on chronic visceral inflammation, hydrothorax supervened, and proved fatal. In one case, a married female, there was inflammation and abscess of the breast. Abscess in the neck, equally annoying to the patient and the surgeon, was a frequent occurrence; but this inconvenience seldom, if ever, arose after general bleeding. Severe epistaxis was controlled, in one case, with difficulty; and the child rallied. Dr. Fothergill, though a humoral pathologist, had the sagacity to guess the true cause of these severe hemorrhages, which point, and not obscurely, to a better pathology. 'It has happened,' says he, 'in this distemper, that hemorrhage from the nose and mouth have suddenly carried off the patient. I have heard of the like accident from bleeding at the ear; but these fatal discharges most commonly happen after the patient has been ill several days; and it seems more probable, that they proceed from the separation of a slough from the branch of an artery, rather than from the fulness of the vessels, or an effort of Nature to relieve herself by a salutary crisis.—This, I find, was also Heredia's opinion, who considers a discharge of blood, either from the mouth or nose, as a sign of the utmost danger.'* Hemorrhage from the intestines attacked another child after the fever had declined; and its removal was followed by purulent ophthalmia. This patient had not been visited professionally in the earlier stages; and it eventually sunk under reiterated irritation. Chronic dysentery appeared in one solitary case, after the subsidence of fever. In another, ulceration and sloughing of the throat, as well as of the leechbites externally, from pure constitutional and strumous debility, continued long after the fever had vanished, as late, indeed, as the fourth week; when an extension of the ulcerative process to the larynx destroyed life by suffocation. The child had been moderately depleted by leeches only; and ammonia, quinine, and wine, were employed in aid of its enfeebled powers."

In the first variety the treatment was, on the whole, successful. In the third and fourth varieties, "the fatality was painfully great, though eventually lessened by a freer use of the lancet." Several cases are recited by Dr. Sandwith, in illustration, for which we refer those of our readers who have not the Edinburgh Journal in hand, to the present pamphlet. We hope soon to have an opportunity of again ushering Dr. S. to the attention of our readers.

---

* Fothergill's Works, p. 213.

Elements of Bedside Medicine and General Pathology; or general Disease—Discourse, &c. &c. &c. By J. Stewart Thorburn, M.D. Octavo, pp. 437, Vol. I. Longman and Co. Highley, Nov. 1835.

This is one of the most extraordinary volumes we have ever met with. It is utterly insusceptible of analysis—and we are never very much addicted to mere criticism. It is a kind of granary filled with extractions or digestions rather than selections from various authors who have written on semeiology, pathology, clinical medicine, &c. in France especially. The works, therefore, of Landre-Beauvais, Chomel, Ratier, Rostan, Serre, Cayol, Dance, Bricheteau, Dubois, &c. &c. have been laid under contribution, and much of their valuable contents have been distilled through the English author's alembic, for the benefit of the English student.

"By quarrying the chief facts and views contained in the subjoined range of select authorship, and working them up in accordance with a digested plan, I trust I have succeeded in architecturing a literary edifice, which may be considered, in some degree, worthy of being occasionally tenanted by the mind of the clinical student of this country." —*Pref.*

The materials, as we before observed, are very valuable; but we suspect that many will find fault with the architecture, and not a few will be unable to comprehend the "digested plan," on which the author has proceeded. No doubt he perfectly understood this plan himself; but it requires no small perspicuity in an author to convey a *novel* plan to the generality of readers. This difficulty, we fear, Dr. T. will find in the sequel; and we are sorry for it, because the book contains a mine of useful knowledge—the parts, we must candidly confess, connected by materials not well calculated for the purpose designed—the instruction of the *young* student. We conceive, indeed, that the book is far better calculated for advanced students—or even actual practitioners, than as a guide to the pupil when commencing his course of clinical instruction in the wards of an hospital. The first years of medical study ought to be chiefly occupied in *observation,* rather than *reflection.* He ought first to lay in the materials, before he begins to think upon them, or arrange them into doctrines or systems. There are a number of speculative subjects introduced into this work, which are calculated to abstract the attention of the student from actual facts before his eyes, and bewilder him in metaphysics. The second part of the volume consists of an " exposition of the medical creeds of materialism and vitalism, &c. &c." This occupies 156 pages of letter-press, and embraces, among other recondite matters, the question, *how man was first formed?* Now we seriously ask Dr. Thorburn whether he thinks the *student,* who is overwhelmed with lectures, and his whole time occupied in acquiring elementary knowledge, can possibly dive into these mysterious subjects, without losing the substance, while hunting after the shadow?—Should the work ever come to a second edition, we advise the author to strike out three-fourths of the matter, retaining nothing but those portions that are adapted to aid the student in acquiring *elementary* and *practical* information—the only species of knowledge which he ought to aim at, while in the schools. When he leaves the lecture-room, the hospitals, and the dissecting-rooms, to search after private practice, *then, and not till then,* would we recommend Dr. Thorburn's work, in which he will find a vast magazine of valuable information, mixed up with boundless speculations that will exercise the utmost stretch of his thought and imagination. We have reason to know, that the author is a young physician of great promise, and unwearied industry. When his judgment becomes a little more matured, so as to keep his genius in check, he will produce a less erudite, but a more useful volume than that which is before us. We recommend it, finally, to the practitioner-student, and not to the school-student.

---

### INDUCTION OF PREMATURE LABOUR.

We select the following melancholy case from the 42d and 43d parts of Dr. Davis's excellent and economical treatise on obstetric medicine.

*Case.* " In the summer of 1819, Mrs. P. became the subject of a profuse hæmorrhage in the earlier part of the eighth month of her first gestation. She was at the time occupying a temporary residence at Richmond in Surrey. The amount of the hæmorrhage was so great as exceedingly to alarm her, and to induce her forthwith to return to her house in town. Having previously engaged the author to attend her in her expected confinement, she sent for him immediately upon her arrival in London. The orifice of the uterus was found on examination but slightly dilated, and as rigid as if no hæmorrhage had been sustained, although it was described as amounting to nearly A GALLON of blood. This statement, however, it is to be presumed, must have been much exaggerated. The circumstances of the case were such as greatly to have alarmed her husband and the members of her own family, and it was suggested that it might be desirable to obtain a consultation. It was accordingly arranged that a consultation should be held on the evening of the same day. It was attended by the late Drs. Sims, John Powell, and by the present reporter of the case. After each of the party had satisfied himself that he could feel a small portion of the placenta attaching to, and even in the form of a slender stringy substance protruding a little way through the orifice of the uterus, it was resolved to recommend the operation for the induction of premature labour without delay. This resolution was no doubt partly formed on the exaggerated representations given of the amount of the hæmorrhage which had occurred at Richmond. Inasmuch as the author had been pre-engaged to attend the patient in her confinement, the duty of operating of course devolved on him.

The operation was performed in the presence, first of one and then of the other of his friends who had assisted him in the consultation; and he believes with as much slowness and caution as could be well practised. It indeed occupied between four and five hours in the performance; which is itself at least a presumptive evidence that the precautions usually observed in such cases were not neglected. After effecting the dilatation of the orifice of the uterus, the presenting ovum was found unruptured, and the lower extremities of the child, which could be felt through the membranes, were brought down without difficulty. After the removal of this child, which was alive and vigorous, although unusually small for its period of supposed uterine growth, it was soon discovered that there was another in the uterus. The membranes of the second child presented as in the other case, and the second birth was completed in about a quarter of an hour after the first. The double placenta, for such was the case, was thrown off in the course of about another quarter of an hour, and no hæmorrhage was sustained after its removal. Both children were alive and vigorous. It was not however supposed they could long survive; and they accordingly died in the course of the forenoon of the day following. The mother was considered next day to be doing pretty well, after having enjoyed a TOLERABLY good night: she nevertheless complained of some headache and fever, and had had little or no lochial discharge. The skin was rather harsh and dry; the tongue was white and loaded; and the patient had drunk very freely of diluent fluids. Her pulse was perceptibly excited: but what she most complained of was a painful throbbing, which she referred to the small of the back. There was also some tenderness of the hypogastric region; for the relief of which, a dozen leeches were ordered to be applied to the part affected. A general bleeding was not advised, in consideration of the very great quantity of blood which had been already lost. Mrs. P. was visited daily till the morning of the fifth day after delivery; when all the gentlemen who had previously met in consultation were sent for in great haste on account of an alarming hæmorrhage with which she had been recently visited. To make short of the account, the patient was found moribund upon the arrival of her medical attendants.

The reader may readily conceive the distress of mind which this unfortunate termination of a case so interesting occasioned to the author; since he could hardly conceal from himself the probability that it had arisen from rupture of a part of the orifice of the uterus, in consequence of too much force applied to it, notwithstanding the extreme caution with which the operation of premature delivery had been performed.

Permission having been obtained to inspect the body after death, it was some satisfaction to find that no such rupture as had been apprehended had actually been inflicted. The cause of the hæmorrhage however became sufficiently manifest. The long-continued bearing incident to the introduction of the hand had produced contusion, inflammation, and suppuration of the os uteri; and a portion of its tissue, about the diameter of a sixpence, had sloughed off, and left behind it a deepish ulcer of the same extent. On examining carefully this part, several considerable branches of arteries were found in the depth of it, open to the day: and thus was rendered evident the cause of the fatal hæmorrhage."

---

### Mr. Middlemore on Pterygium.*

Mr. Middlemore is favourably known to the profession by several interesting and useful papers on the diseases of the eye: the contribution before us merits notice.

Pterygium, as Mr. Middlemore observes, is a morbid growth of a triangular figure, generally commencing at the inner canthus of the eye, at that part of the conjunctiva immediately around the semilunar fold and lachrymal caruncle, its point or smaller ex-

---

* Prov. Med. and Surg. Transactions, Vol. III.

tremity being situated towards or upon the cornea, and its base directed towards the periphery of the eye-ball. This is its ordinary, but not constant situation. For not only may it be seated on some other part of the eye-ball, but it may form on different parts at once, and the pterygia converging on the cornea may utterly obscure the pupil. It is not uncommon to observe two pterygia upon one eye—one arising from the inner, the other situated at the outer canthus.

"Perhaps," says Mr. M. "I may be allowed to state upon this subject, as the result of my own experience, that the frequency with which pterygium occurs, as respects the various parts of the eye, takes place in the following order: first—the formation of a pterygium upon each eye, arising from the inner canthus; secondly—the occurrence of two pterygia upon one eye, one arising at the inner, and the other at the outer, canthus; thirdly—the formation of one pterygium alone, either at the outer canthus, or at the upper or lower part of the eye-ball, but not at the inner canthus. The occurrence of four pterygia upon one eye, the points of which have met upon the cornea and completely obscured the pupil, constitutes, in the opinion of the celebrated Scarpa, the pannus of the ancients. I have only witnessed one case, and that very recently, in the eye of Joseph Burley, a patient now under my care at the infirmary."

Mr. Middlemore agrees with Scarpa in believing that the triangular form of pterygium, is dependent on the increasing strictness of adhesion between the sclerotica and conjunctiva, as the latter approaches the margin of the cornea, and between the conjunctiva and the cornea, as the former advances to the centre of the latter. He disagrees with Scarpa in thinking pterygium a conversion of the conjunctiva, but conceives it to be a growth under it. Having premised this we may present our author's description of pterygium.

"The first appearance of pterygium is indicated by a few enlarged conjunctival vessels, proceeding from the inner canthus, (I will assume that to be its situation, for my present purpose) and running in a direction nearly parallel to each other; at the same time, there is an appearance of increased thickness in the conjunctiva, just as though a fine web, or film, had been spread upon its surface. In a short time, there is seen a small reddish deposition, situated at about two or three lines from the corneo-sclerotic junction, and the vessels proceeding to, and terminating in it, begin to assume a more definite and distinct arrangement; by very slow degrees this deposition near the cornea becomes more apparent, increases in size, and approaches nearer to the cornea, until it reaches the line of union between the cornea and sclerotica, where it is seen to project above the corneal margin. The vessels proceeding to it, and constituting part of its volume, now become more numerous—the texture in which they are placed increases in density and opacity—and its outline is rendered perfectly distinct by its elevation above the surrounding portion of conjunctiva; it is also quite moveable, and may be raised more or less extensively from the sclerotica, according as its connexion with the texture beneath may be merely *filamentous*, or by a greater extent of more intimately connected surface. At this stage the nature of the disease is rendered quite evident; there is, in short, a red triangular deposition beneath the conjunctiva, which is gradually acuminated towards the cornea, where it rises in a distinctly elevated projection around its margin.

The disease may remain for a long period in this situation, before it makes any discoverable progress upon the cornea; but, after a time, if no measures be employed to impede its growth, it continues its course, acquiring thickness and strength as it advances, until it appears like a triangular muscle, and may extend (gradually tapering as it proceeds) to the centre of the cornea, particularly if a similar pterygium be advancing from the opposite side, when its point is much finer than in those instances in which no opposing pterygium exists."

Passing over many pages of diffuse remarks, we arrive at Mr. Middlemore's division of pterygium into three varieties, viz. 1, the membranous, or *pterygium tenue,* which is merely a thin semi-transparent layer, in which the course of the vessels may be distinctly observed;—2. the fleshy pterygium, or *pterygium carnosum,* which resembles, in outward appearance, the fibrous structure of a muscle;—3. the adipose pterygium, or *pterygium pingue,* which is chiefly of a white, soft texture much resembling common fat. This latter variety of pterygium is, however, much less common than the two former.

In a case of the sarcomatous pterygium which had gone to some extent, and in which Mr. Middlemore removed the morbid growth by the knife, he found the cornea beneath it opaque, and vascular, and thickened. He therefore cautions the young surgeon against anticipating much success in similar cases, in opposition to the opinions expressed by many writers on ophthalmic surgery. He observes, too, that in old cases of large sarcomatous pterygium, when the disease has arrived at the centre of the cornea, it spreads laterally, so as to acquire a very obtuse extremity, and that in this way it may obscure nearly the whole of the pupil, and destroy all useful vision. It does not proceed from the centre directly across the pupil; it does not progress in a direct and continued course; but covers the greater part of the cornea by its expansion laterally, when its direct progress has been arrested by its arrival in its centre; so that, eventually, nearly the whole of the cornea becomes concealed and obscured.

The prognosis of pterygium is simple enough. The earlier it is taken the more curable it is, for if it has lasted long and extended far on the cornea, it has probably adhered to and occasioned serious alterations of that tissue. For the diagnosis of pterygium the reader or the student has only to refer to the description.

We arrive at the treatment.

We may sometimes, says our author, arrest the progress of pterygium by the use of astringents and stimulants; and the cases in which they are most usefully employed for this purpose, are, when the pterygium is small and of recent origin, and is actually increasing in size. The common zinc wash, or the nitrate of silver or sulphate of copper drops, in the proportion of two or three grains to the ounce of water, are the best remedies for this purpose; and the patient may be directed to allow a little of the one or the other of these applications, as may be preferred, to be dropped upon the pterygium, from a common capillary tube, two or three times a day. If, by these means, its increase can be checked, then their use may be suspended, (and this is more particularly necessary when the nitrate of silver drops are employed on account of their tendency to tinge the conjunctiva) for it not uncommonly happens that, when once the progress of pterygium is arrested, it will not again increase, or will at least remain stationary for a long time; still, if at any future period it should evince a disposition to enlarge, the same practice may be again employed.

Escharotics were much used, but they are mischievous; and scarifications have been employed, but they are inefficient. Partial excision is the simple and effectual remedy. Various modes and instruments have been adopted. The following is Mr. Middlemore's.

" The patient should be requested to lie upon a table or sit upon a chair, and, having the eyelids well separated by an assistant, so as to expose the whole of the globe, (which should be steadied by the firm pressure of the index and middle finger on each side) the operator should grasp the pterygium with a pair of hooked forceps, and having raised it from the sclerotica, pass the probe-pointed blade of a *well-made* pair of scissors underneath the elevated portion, midway between its base and the margin of the cornea, and having divided that portion, as far as its union with the sclerotica will permit, he turns the scissors to the opposite side, which should be also divided in the same way; with a fine scalpel the pterygium may be then dissected and detached from the sclerotica and the cornea by a succession of careful incisions, taking care that the flat side of the scalpel is directed towards the globe of the eye, so that its cutting edge shall be directed rather away from, than towards it.

If an incision be made upon the pterygium from its external side towards the globe, the sclerotica may be either cut or injured, or much unnecessary pain excited by partially dividing the pterygium in various places; or if, with a view of preventing the necessity of making several incisions, and with the intention of severing the whole of the pterygial production at once, the operator, in the plenitude of his courage and dexterity, makes one free section, the eye-ball may be either slightly incised or completely punctured; and if the scalpel be placed underneath, and the pterygium cut from within outwards, as Mr. Guthrie (p. 142) recommends, it may be raised, indeed, nearly half an inch from the eye-ball, and very probably *torn from* sclerotica or cornea, rather than divided. And this is not merely a theoretical objection, but a matter of the most probable occurrence; and I have myself seen that portion of the conjunctiva situated at the upper part of the eye-ball, and which forms its point of reflection, pulled quite downwards, and nearly the whole of the conjunctive membrane raised, and absolutely *stripped* from the sclerotica and the eye-lid, during an attempt to detach a pterygium situated at the upper part of the eye-ball, in the mode recommended by Mr. Guthrie.

I may further remark, that the scissors employed ought to be rounded at the extremity of that blade which is passed beneath the pterygium and pushed against the conjunctival fold which connects it with the sclerotica, lest it should penetrate or otherwise injure the eye-ball; and the same precautionary remarks applies to any, and every, instrument which it may be considered necessary to pass beneath a pterygium for the same purpose, as it is quite impossible to prevent, in every case, by any care and precaution the operator may employ, the sharp point of an instrument from hitching upon or puncturing some important part in an unsteady eye, the muscles of which are contracting (as they will do under the excitement of an operation) spasmodically."

After the operation, the usual means should be adopted to exclude light, keep the eye-ball clean, and prevent adhesion of the eye-lid to it. After the excision of the pterygium, small portions of loose ragged conjunctiva may remain, as it may be difficult to take away the whole of the pterygium at once, and in either of these cases it is desirable to remove the loose portions by means of the curved scissors, at a subsequent operation, if they do not contract under the use of astringent applications; at all events, it is not right to attempt to destroy them by the aid of strong caustic and escharotic substances.

Such is Mr. Middlemore's paper on pterygium. Its chief, perhaps it only fault, is its diffuseness. This, unfortunately, is too generally the case with the papers contained in the Provincial Society's Transactions. Were they put into a hydrostatic press, the diminution of bulk that would ensue would occasion a proportionate increase of value.

---

CASE OF GREAT ENLARGEMENT OF THE ARTICULAR EPIPHYSES FROM RICKETS. By THOS. BRAYNE, Esq. of Banbury.

Mr. Brayne, who appears to be a very intelligent surgeon, has related the following case in the last volume of the Provincial Society's Transactions. He has added to his narrative a sketch of the unhappy patient, which confirms whilst it illustrates the description.

*Case.* T. P., æt. 7. He was born of healthy parents, whose seven other children, though of strumous diathesis, were tolerably healthy also. In three or four weeks after birth, the subject of the case became affected with diarrhœa, and with slight stiffness and enlargement of the right elbow. The diarrhœa continued unabated up to the year preceding this report. It then greatly decreased, it was attended with prolapsus ani. At the age of seven months he began to waste in his limbs and body; the knees then became enlarged, and after them the wrists and ankles became so too.

His state at present is thus delineated by Mr. Brayne. The knees and elbows are anchylosed nearly at right angles. His countenance is sallow and rather vacant; but there is often a sparkling in the eyes, and a quickness of attention, when he is addressed, as well as a pertinence in his answers, which show at once that he possesses and has acquired much mental power; and which is in nothing more manifest than by the most perfect resignation, and often by the recommendation of it to his younger brothers, when they are peevish and discontented. His skin is dry and unhealthy, and there are many small spots of a scaly eruption, like the psoriasis guttata of Willan, scattered over his forehead, neck, and shoulders. His voice is squeaking and discordant, as though uttered through a reed, with a peculiar dry, sibilous tone, which gives one the idea that there is something imperfect in the vocal caitilages or membrane. There is, however, no external hardness, or enlargement about the throat, either of the laryngeal cartilages or of the thyroid gland. The shoulders are rather high; the ribs compressed laterally; the sternum prominent; and the extremity of the ensiform cartilage slightly everted. The abdomen is considerably tumefied. The right knee measures one foot, seven inches in its greatest circumference, and is as large as the head, within one inch. The relative proportion of the articular end of each bone which enters into the composition of the joint, as well as the situation of the patella, can be discovered by careful examination, and they all seem enlarged in nearly the same degree. The surface feels hard and unyielding, and large veins ramify over it, adding to the dark red colour which the skin has acquired from long distention. The integuments of the interstices between the bones are very slightly œdematous. The left knee measures only one foot, three inches, in circumference. The elbow, wrist, and ankle-joints are also greatly enlarged. The elbows are also so much anchylosed, that all flexion and extension are destroyed—so much so, that he can only reach his lips with the ends of the fingers of the left hand, and this with the greatest difficulty. The hands are only the size of those of a child of two years old, and the fingers are clumsy and ill-formed.

The lithographic sketch accompanying the paper displays these deformities very prominently. Mr. Brayne makes some sensible remarks on the preceding case. It was clearly one of rickets; at least the enlargement of the articular symphyses evidently depended on that state of constitution which gives rise to rickets. A slighter degree of the same thing is not rare. We have at present under our care a young lady, in whom the articular epiphyses of each tibia are enlarged. She has a disposition to rickets. This enlargement of the epiphyses of the tibia is not unfrequently combined with a very troublesome curvature of those bones outwards. The curvature appears to take place at the junction of the epiphysis and shaft. The best means of counteracting it is by taking the weight of the trunk off the lower limbs by means of proper instruments, and strapping the convex tibiæ to an upright steel rod, which extends from the boot to the inside of each knee. Of course there is a jointed rod on the outside, leading up to the pelvic girth. Yet the complaint is difficult of management.

Mr. Brayne points out the paramount necessity of attending to the state of the secretions in cases of rickets. This is, indeed, of great importance. Yet, when the secretions are moderately re-established, we must look to good air—cold bathing—good diet—and tonics, especially steel and sarsaparilla—we must look, we repeat, to them for the principal amount of benefit.

**Remarkable Disease of Stomach and Colon. By Dr. J. Johnson.**

A gentleman of rank, aged about 50, thin, and below the middle size, of remarkably active habits, and free from any disease or disorder, except torpid bowels, which he much neglected, was subjected to considerable mental anxiety in the year 1835. This, as is usual, disordered the functions of the stomach and bowels, rendering the constipation more obstinate, and occasioning what are vulgarly but indefinitely called—bilious symptoms. In this state he was one day, from choice rather than necessity. exposed to a long-continued fall of rain on the box of a stage-coach, by which he was so much chilled that he experienced an actual rigor, and was advised by the coachman to drink a wine-glassful of brandy while the horses were changing. This restored warmth, and he reached London without further molestation. This was about three or four months before his death, and he dated the commencement of his fatal illness from this gratuitous exposure. Yet, after this period, he hunted in the very worst weather, and could bear fatigue that exhausted young and vigorous huntsmen. To constipation of bowels was now added a new symptom—the inability to take his usual quantity of food, and, occasionally, the rejection of part of the ingesta after an hour, two hours, or three or four hours from the time of eating. The frequency of vomiting gradually increased; and the capacity of the stomach for the reception of food proportionally diminished. The obstinacy of the bowels was also a regularly increasing evil. In the month of September, of the year 1835, an obstipation, amounting to a complete stoppage took place, accompanied by augmented irritability of the stomach, so that the case was considered serious, and several medical men were joined in consultation, when various means were employed, and several days elapsed before a passage through the bowels could be effected. It was, however, distinctly averred (and there could be no doubt of its truth) that, at this time, and for three or four days in succession, a free evacuation of fæculent matters from the bowels was obtained, with considerable remission of the symptoms. But it was quite evident then, and for some time previously, that emaciation was advancing with steady pace. This relief of the bowels was only temporary, and the gastric irritability continued. The quantity of food that could be taken was very small, and almost entirely liquid. The gentleman, who was of the most patient and placid temper, felt as if every thing lodged about the cardiac orifice of the stomach, which, however, was a deceptive feeling. The stoppage of the bowels returned, and, after several days consumed in ineffectual attempts to procure evacuations, Dr. Johnson was sent for to the patient on the 20th October. After learning the foregoing particulars, Dr. J. proceeded to make a careful examination. The emaciation was extreme; but the abdomen was not shrunk in proportion. On the contrary, there was some degree of fulness, especially about the navel, as well as tenderness on pressure. No tumor could be detected in the region of the stomach, or in any part of the abdomen. He had vomited that morning nearly a pint of glairy fluid, with some dark-coloured particles. The tongue was furred in the middle, and rather red at the sides and tip—pulse 110—skin dry—and a febrile movement in the evening. The urine was very scanty and high-coloured. As some degree of peritonitis was suspected, eight leeches were applied to the umbilical region, an emollient injection was thrown up, and a grain of calomel was ordered every four hours, without any other medicine. There was no return of vomiting for four days, and then only in a trifling degree—nor did the patient afterwards complain of any pain, except from occasional flatulence. During the two first days of Dr. Johnson's attendance, and in consultation with three highly intelligent medical practitioners in ———, injections were assiduously employed at least three times a day, persevering steadily with the calomel, and employing mercurial frictions with croton oil, occasionally, on the abdomen. Not a particle of fæcal matter could be procured by these means, and it was deemed prudent to avoid all powerful medicines by the mouth, in order not to rekindle the gastric irritability, which caused the greatest distress. The prudence of this plan was doubted, at the time, by several of the patient's anxious friends, who naturally enough reiterated their entreaties to use some *active* means, to rescue the valuable life now in imminent danger. But the patient now passed his time in comparative comfort, sleeping four or five hours every night, and, being sensible of his state, often writing letters of farewell to his friends in the day. Broths and milk were now thrown up, twice a-day, into the bowels. They usually remained some hours, and occasionally they did not return at all. By these, the sense of inanition was removed, or greatly relieved—for he could only take a few spoonfuls of asses' milk, or other bland fluid by the mouth. Dr. J. remained five days in ———, and, upon mature reflection on the case itself, and in full accordance with his excellent medical companions, refused to hazard any experiments that might embitter the last days of the patient's life, and not do any good to the disease itself. The reasons on which Dr. J. and the other medical attendants grounded this passive plan of treatment were these. They considered that mechanical obstruction, the result of disease, existed, which no *active* purgative or other remedy was likely to remove, but which would, in all probability, be greatly exasperated by active treatment. The medical attendants thus subjected themselves to some comments, and considerable responsibility; but they were steady in their resolution, and the event shewed that they were right in their judgment. On leaving the residence of the gentleman in the country, Dr. J. put down the following opinion in writing, and left it in the hands, not only of the medical attendants, but of the family and their friends.

" *B. House, near S——y,*

*Saturday, 24th Oct.* 1835.

After the best consideration which I could give to the case of ———, I have come to the following conclusions:

1st. That anxiety of mind disturbed the functions of the stomach and bowels.

2dly. That there appears to have been originally, and even now, some obstruction to the passage of the chyme from the stomach into the bowels.

3dly. That chronic inflammation has supervened, within the last fortnight or three weeks.

4thly. That an obstruction *now* exists in the intestinal tube, in addition to the original affection of the pyloric orifice of the stomach.

5thly. That there are, in all probability, considerable adhesions among the intestines.

6thly. That there is reason to apprehend sero-purulent effusion within the cavity of the peritoneum.

7thly, and lastly. On reviewing the treatment, before and after I visited ———, I cannot now see any thing that could have been done more or better than what was done.

James Johnson."

After Dr. J. left the country residence of ———, he had a regular correspondence with his medical friends. The same plan was continued, except that the calomel was taken in less quantities as the gums began to get sore. No alvine evacuations could be procured, but the fluctuation entirely disappeared before the death of the amiable patient, which took place without any suffering on the evening of the 1st of November, the complete obstruction of the bowels having lasted at least 25 days!

Dissection.

" Omentum much wasted. The mesentery shrivelled. The internal coat of the stomach healthy, excepting here and there a few patches of petechiæ. The lesser curvature of the stomach appeared like a hard band or cord, which increased in thickness as it approached the pylorus. The whole circumference of this aperture was in this thickened indurated state, extending to some distance along the greater curvature of the stomach. In consequence of this thickening, the orifice of the pylorus was much contracted, but not so as to entirely close up the opening. The pylorus itself was firmly united to the transverse arch of the colon, by the same kind of diseased structure above-mentioned. The adhesion was so firm, that the pylorus and this part of the colon, at first view, appeared like one intestine. They could only be separated by dissection. In consequence of this union, and the diseased and indurated state of the coats of the colon at this part, the canal of the colon was contracted so much as to allow air only to pass, and then when pressure was used. The duodenum was healthy, and so were the small intestines. The caput coli, ascending arch, and part of the transverse arch of the colon, till just before the strictured portion, were enormously distended with gas. The colon measured, at this place, 12 inches in circumference. The gas would not pass through the stricture till pressure was employed. There was also a great quantity of healthy fæces in the caput coli and ascending portion. The descending colon and rectum were healthy, and quite empty. There was no ulceration of the coats of the stomach or bowels. The intestines contained much air, but there was no adhesion—no glutinous matter—no appearance of inflammation or disease, except what is mentioned above. No effusion above an ounce into the cavity of the abdomen. There was a slight adhesion between the liver and colon, near the strictured part. The other abdominal organs were free from disease."

It will be seen by the above dissection, that the diagnosis was right in two out of the four particulars. The obstructions in two different portions of the alimentary canal were the most important items, and on which hung the fate of the patient. Both Dr. J. and the other attendants were very confident, at one time, that fluctuation existed in the abdomen, though they were probably deceived by the accumulation of fluids and other matters above the stricture of the colon, where that bowel was so immensely dilated—and where fluctuation might not easily be discriminated from a general effusion. The existence of chronic inflammation and adhesions was, of course, matter of conjecture, and, as it turned out, the conjecture was erroneous. The main calculations were, however, correct, and they absolved the medical attendants from any censure that might have been applied, by those who advocated the exhibition of heroic remedies. The amiable family of this gentleman gave permission for a *post-mortem* examination, with a view to benefit the living. The case is now published in furtherance of that object. It ought, and it will, prove a lesson to those who consider that obstruction in the bowels is a merely mechanical one, and independent of some inflammatory or organic lesion, of which the obstruction is, in fact, the result, or necessary effect. Excepting, indeed, obstructions from hardened or accumulated fæces, we know of hardly any case where drastic purgatives can be safely used. The history of the case will generally inform us whether there is suspicion of chronic disease being the cause of the obstruction, as in the case here detailed—and where this is the case, we conceive that the mildest means alone should be resorted to.

The adhesion of the scirrhous pylorus to the middle of the transverse arch of the colon, and the participation of the *latter* in the disease of the *former*, are circumstances that ought not to pass unnoticed. We may fairly presume, that the adhesion between the two parts above-mentioned was the result of some temporary inflammation, occurring at a period long before the illness which terminated life. But we may also safely infer, that the scirrhous state of the pylorus was propagated to the coats of the colon (which were now in intimate union with the pylorus), and that thence resulted the thickened coats of the colon and the obliteration of its calibre. We know that scirrhus and cancer are propagated from one tissue to another in the body—from the softest to the hardest parts. Mr. Hooper, of Southwark, lately presented to the London Medical Society a very fine specimen of chimney-sweepers' cancer, spreading from the scrotum to the os pubis, and involving the latter in its destructive ravages. This was clearly the case in the present instance.

The medical attendants on this distinguished patient, had the melancholy pleasure of knowing that the relatives of the victim of an incurable disease were not only satisfied with the judgment of the said attendants, but were grateful, in the highest degree, that the patient was kept in the least possible state of pain, by their abstaining from all violent measures, which would have aggravated the unavoidable sufferings of the dying man, without affording the remotest chance of success. On this account alone we deem the case worthy of record, as a beacon and a lesson for our junior brethren.

---

Desperate Wound in the Neck, in an attempt at Suicide. Communicated by Mr. Daniel Tyerman, of Banbury, Oxon.

[Extract of a Letter.]

" *Aug.* 14*th*, 1834. Mr. —— æt. 35, a gentleman of robust make and constitution, while under the influence of delirium tremens, in a moment of inincreased excitement, seized a worn-down dinner knife, and plunged its blade into the right side of his neck, the point entering beneath the lobe of the ear and behind the angle of the jaw, severing the external, and probably from the extent of the wound, the internal carotid arteries, and internal jugular vein. The point of the instrument entered the pharynx and struck upon the spine. A line drawn from midway, between the lobe of the ear and angle of the jaw transversely across the lower lip, would indicate pretty nearly the direction of the wound, the external orifice of which was inclined obliquely downwards and forwards, and was about one inch and a half in extent.

Being in a neighbouring apartment, Mr. Thomas Norris and myself were immediately summoned, and we found the patient sinking from loss of blood into the arms of an attendant; blood in profuse quantity gushing from the wound and from the mouth, the lower lip being drawn to the left side.

As he lay in the recumbent posture, I introduced my left fore-finger into the wound, its point passing into the pharynx, a spasmodic motion of whose fibres was plainly felt, also a notch inflicted by the knife in the anterior common ligament covering the body of the second cervical vertebra. The internal carotid was plainly pulsating beneath the finger. At this time the patient, in efforts to spit out the blood which was flowing into the mouth, depressed the jaw, the angle of which being exposed by the knife pressed sharply on the finger. The hæmorrhage was much restrained by this proceeding, and blood still flowing I made pressure with my right hand upon the common carotid trunk. Notwithstanding these exertions, blood continued to flow until a large plug of lint was quickly inserted deeply into the wound upon my right fore-finger, the left being withdrawn. The hæmorrhage was now nearly restrained, a few drops only oozing by the side of the plug.

I proposed immediately operating on the common trunk. Mr. Norris assenting to the proposal, with his able assistance, I secured that vessel below the omo-hyoid muscle, Mr. N. being in readiness to make pressure upon the artery as low as possible in case of a renewal of the hæmorrhage. No blood was lost in the operation, and the ligature commanded all hæmorrhage. The descendens noni lay rather to the right of the sheath just about to cross it, and a large cardiac nerve ran down upon the artery in contact with its coats. The vein was not distinctly seen in the operation, being collapsed, this state indicating the great quantity of blood lost, or its division above by the knife. No complete fainting took place, notwithstanding the enormous quantity of blood lost, which could not be estimated at less than sixteen pounds. Not more than a minute could have elapsed before our attendance, and about a pound and a half of blood flowed from the wound and from the mouth in our presence. The patient was carried to bed after lying an hour or two upon the floor, and having somewhat rallied—the pulse being 60 and feeble.

Mr.Norris and I having consulted, we decided upon placing him under the influence of opium and gave liq. op. sed. gtt. x. 6tis horis. The bowels had been freely opened in the morning by enema and purgatives. He lay in a half-dozing state during the remainder of the evening. Mr. Callaway of Guy's Hospital saw the case towards night, and corroborated our plan of treatment.

Aug. 15th, morning. Has dozed much, but obtained scarcely any sound sleep. Pulse 96, small, with hæmorrhagic hardness—tongue dry, glazed, and when moved the right side is sore and painful. Thirst—skin warm and perspirable—deglutition difficult, but performed without necessity for the tube—no hæmorrhage. Small coagula have been spit from the mouth. Sero-sanguineous oozing from the wounds. Sensation and warmth diminished upon the right side of the face. Milk and arrow-root diet. Rept. med.

Midnight. Has slept imperfectly for an hour, with startings and half-closed eye. Respiration accompanied with a guggling noise. Pulse 100 softer and fuller, with slight deviations in its character. Skin perspirable. Deglutition performed with difficulty, but not followed by pain.

16th, Morning. Great restlessness during the night. The plug of lint causing much uneasiness and difficulty of breathing was gradually removed from the wound this morning without hæmorrhage—it is covered with well-formed pus, and the surfaces of the chasm are granulating healthily. A pledget of lint to be passed daily into the wound upon a director, a pad placed under its orifice; poultice over the orifice. Deglutition and respiration facilitated by the removal of the plug. Bowels not open—tongue dry. Enema decoct. avenæ. The same diet—rept. opium.

Midnight. Has slept for three or four hours, and starts on awaking. Urine passes involuntarily in small quantities, and has a strong ammoniacal odour. A pain in the stomach of which he complained was removed by small quantities of dilute brandy. Pergat.

17th. Has improved, and has slept for several hours. The wound discharges freely, and deglutition much less difficult. Omit opium.

18th. Tongue becoming moist and cleaning at the edges—much less thirst. The bowels being torpid, Rh. p. viii. cal. gr. ii. were given, which freely operated, after which the pulse fell from 100 to 72. A fainting fit occurred at noon during slight exertion in changing the bed. Urine secreted in larger quantities, is retained, and has completely lost its ammoniacal odour. The opium has been repeated since he showed some increased irritability—also small quantities of dilute brandy. Port wine with arrow-root.

19th. Going on well, though much excitement and restlessness continue. Appetite pretty good; not yet able to masticate, as the lower jaw can be only slightly depressed. Deglutition performed without pain. Pergat.

21st. Considerable restlessness at night, but he appears improving.

23rd. From some excitement to-day the pulse is 84 and slightly jerking, and he feels some constriction about the glottis on taking a deep inspiration, which was relieved. Sp. æth. sulph. comp. ʒj. liq. op. sed. gtt. xij. hora somni sd. Rept. opii.

25th. The ligature of the common carotid separated to-day—the deep wound is fast closing. Restlessness continues at night.

26th. Mastication and deglutition performed with tolerable facility, and depression of the jaw is performed to nearly its natural extent. Porter Oj. a day—light pudding.

29th. More animal food than ordered was given, and slight febrile action was induced. An opiate, and the calomel purgative were given.

30th. Bowels well open—the deep wound fast closing, it being only ¾ of an inch deep.

Sept. 1. Is able to sit up for three or four hours, and walk up and down the room. Bowels open — appetite good, though some restlessness at night continues. Fast recovering strength. From this date, complete and rapid recovery took place—the countenance for a long time however remaining exsanguine. The cicatrix of the wound was depressed, and extended from the edge of the sterno-cleido mastoid muscle, as it is passing to its insertion, to the angle of the jaw. The lower lip still remained drawn to the left side, and articulation in consequence somewhat imperfect."

---

### Scandinavian Contributions to Practical Medicine.

[From the German.]

In the 44th and last volume of Rust's Magazine, we notice, under the title "Contributions to Practical Medicine and Surgery, collected from Scandinavian Sources," a series of cases and observations, communicated by Dr. J. F. Neverman, of Plau in Mecklenburg. These "Contributions" possess a twofold interest; firstly, they are in themselves, for the most part, good and valuable; and, secondly, they afford some insight into the state of medical science in Denmark—a subject with which, we dare to say, the generality of our readers are but little acquainted.

For these reasons we will overset, as the Germans have it, a few of the most important among them, commencing with a paper by Dr. Stenberg of Vallö, "On the Employment of Blood-letting and Emetic Tartar in Intermittent Fever."

"In April, 1831, intermittent fever again began to prevail, presenting usually the tertian type, more rarely the quotidian, and less frequently still the quartan. As in the preceding year, the treatment consisted chiefly in the exhibition of Peruvian bark and quinine, by which the disease was removed without much difficulty; but the relapses were frequent. Many, therefore, had recourse to quacks and empirical remedies, and the frequent consequences were a train of morbid phenomena which indicated more or less serious affections of the thoracic and abdominal viscera. The more frequent of these secondary symptoms were those of dropsy, and of indurations of the liver and spleen, dyspnœa, cardialgia, &c. Under these circumstances, I determined to make a trial of the method proposed by Reich,* with which I had lately become acquainted, and which this physician has long practised with distinguished success. Indeed, he asserts that secondary symptoms, (which he regards as the result of neglected inflammations) and that relapses are, in his practice, of very rare occurrence. At first I experienced much opposition on the part of the patients, who complained particularly of the nausea, vomiting, and purging produced by the mixture; when, however, the beneficial effects became apparent, all murmurs of disapprobation were at once silenced. The following are the results of my experience in this mode of treatment.

During a period of three months, I subjected 109 individuals to the plan recommended by Reich; not, however, rigorously adhering to it, as he appears to me to push the bleeding too far, and to give the emetic tartar in too powerful doses. My plan was to open a vein during the paroxysm, preferring the cold stage, or the period immediately preceding this. I never bled the same patient more than three times, and but rarely more than twice. During the paroxysms I gave no medicine, but in the interval between them, I directed a mixture to be taken composed of two drams of sal ammoniac, and from eight to twelve grains of emetic tartar dissolved in eight ounces of water, the dose being a spoonful every two hours. In the case of children, in the place of bleeding, I applied leeches over the cardiac region. By this treatment the hot stage was shortened in a marked degree. At the first bleeding the blood never presented an inflammatory crust,

---

* Grundlage der Heilkunde. Ein Spiegal für Aerzte. Berlin, 1828.

though at the second and third this appearance was frequently observed. In many cases after venesection, and copious evacuations *per anum et per oram*, the disease still continued, and was only completely subdued after a second, or even a third bleeding; in perhaps the half of the patients, the fever, though much mitigated, resisted a perfect cure by these means, but—now it was that I was enabled, almost without an exception, to cure it with a smaller quantity of bark or quinine than before; a decoction of six drams of bark in seven ounces of water being now found sufficient. As far as my observations extended, relapses were but little diminished in frequency; when they occurred, I did not again take away blood, except in the case of plethoric individuals, and then once only, but treated them on the ordinary plan with bark or quinine.

It is an objection to this mode of treatment, that after the removal of the febrile symptoms, a state of languor and debility of long continuance remains; on the other hand, it has the signal advantage of obviating those secondary affections of the thoracic and abdominal viscera above noticed. I feel myself in some way peculiarly entitled to decide on the best methods of treating intermittent fever, having suffered myself for many years from severe attacks, occurring almost every spring or summer. I have personally tested the efficiency of many plans, and amongst others, of the one now under consideration. When bled during the hot stage, the sensations I experienced were extremely agreeable. After two venesections, some traces of periodic fever still remained; these, however, quickly disappeared under a short use of decoction of bark. The consequent languor and debility were of longer duration than usual, but throughout the whole year, I felt an unusual lightness in my body, and was almost entirely free from the feeling of oppression between the chest and abdomen, from which, when recovering from former attacks, I always suffered for many months.

In children, Reich's mode of treatment appears less decidedly beneficial, which surprised me the more, because, in these cases, he particularly lauds its efficacy.

In conclusion, I must observe that I do not blindly and unconditionally follow this practice, and that there are many cases to which I do not consider it applicable; but I cannot and dare not deny, that in adult plethoric subjects, in whom the paroxysms are violent and frequent, and in those suffering from congestions of the thoracic and abdominal viscera, it is eminently beneficial, and under these circumstances, I shall continue unhesitatingly to employ it.

*By Westergard, district surgeon at Kjöge.*

It having fallen to my lot at the commencement of the year 1830, to treat a crowd of patients affected with intermittent fever, of whom many had suffered frequent relapses, and presented symptoms of *obstructio viscerum*, I resolved to make a trial of the method recommended by Reich, who treats this disease as a kind of pneumonia with bleeding and tartarized antimony. The first patients, on whom the experiment was made, were children from the workhouse of this place; many of these had long suffered from a tertian intermittent, which, although removed by emetics, sal ammoniac, bark, sulphate of quinine, ergot of rye, (mutterkorn,) or other remedies, recurred repeatedly after a lapse of three or four weeks. In these patients, who were between 10 and 16 years of age, subsisting on a spare diet, and of a habit of body far from plethoric, I opened a vein each time that a paroxysm occurred, preferring, if circumstances permitted it, the hot stage, and abstracting from four to eight ounces of blood. After the first venesection, and at the termination of the paroxysm, I administered a mixture composed of eight grains of tartarized antimony dissolved in eight ounces of distilled water, in the dose of a half or one table-spoonful every two hours, and I continued this medicine many days after the disease had disappeared. The patients were also directed to remain in bed, and to be restricted to a low diet.

These my first experiments, about 20

in number, were crowned with remarkable success: in some of the cases, the paroxysms did not return after two venesections, more frequently three were necessary, but it very rarely happened that the fourth bleeding did not remove the disease completely. Only in a weakly boy, aged 14, who had suffered from repeated relapses, and had symptoms of hepatic obstruction, did I find it necessary to practise a fifth bleeding before the disease gave way. This boy, in the course of two or three weeks, regained his health and strength, and after a lapse of nine months, still retained them unimpaired. All these patients, together with 40 others, residing in the town or country, whom, at a later period, I subjected to a similar treatment, perfectly recovered their health, and not one, as far as I know, experienced a relapse, or suffered from any consequent secondary affections.

Patients between the ages of 10 and 60 years, I treated in this manner with similar success. From middle-aged adults, I abstracted the first three or four times, at each venesection, about twelve ounces of blood; in succeeding bleedings, if these were necessary, not more than eight ounces; though if a decided effect were not produced after the first or second time, I did not scruple to take away from four to eight ounces more. I have had many cases in which a single venesection cured the disease, and many in which it was reremoved after the second. In two patients thus cured, one of whom had been once and the other twice bled, the fever returned on the 21st day; each was bled to twelve ounces, the effect of which was that in both the disease was at once effectually checked. Experience has convinced me that, to effect a lasting cure, the patient should be bled in every paroxysm, however light it may be; for if this is not done, the disease in a few days regains its original power. In two cases, I was compelled to bleed six times; the patients had had many relapses, and, for a long period, had been under treatment with bark. Rarely did any considerable degree of debility follow the loss of so much blood; but, on the contrary, the patients after the first or second bleeding felt better, the tongue was cleaner, and when the fever ceased, they quickly regained their former strength and health.

I bled in the hot stage; and when the attack was a severe one, and the pains in the head and chest violent, the patients were so greatly and so immediately relieved, that they themselves often earnestly requested a repetition of the venesection in the succeeding paroxysms. In a few individuals whom, on account of debility, I did not consider it advisable to bleed again, the disease was speedily removed by a decoction prepared with one ounce of bark, half an ounce of valerian, four grains of emetic tartar, and eight ounces of water. In the case of children under eight years of age, leeches were substituted for venesection, but not always with success; however, in many I succeeded in removing the disease by a solution of emetic tartar.

I am aware that many physicians are unfavourable to this method of treatment, but I firmly believe that it deserves attention, and we ought not to reject it without a fair trial. During a period of nine months, in which I treated 60 patients, I became convinced of its superior efficacy; no other practice so quickly removes the disease, and none other so completely obviates relapses, and secondary organic affections."

Dr. Neverman remarks, that the foregoing observations, for which, he says, we are indebted to two excellent physicians, and observers of nature, were communicated to the Royal Board of Health at Copenhagen, and by that body directed to be printed. Bleeding in intermittent fever has been long advocated in this country by Dr. Mackintosh of Edinburgh, and those of our readers, who have perused the account given by that eminent physician of the effects of this practice, will not fail to remark the accordance of his results with those obtained by Stenberg and Westergard. It appears that bleeding may be practised with perfect safety and perhaps equal benefit, in the hot as in the cold stage, a fact which sadly invalidates the aphorism of old Celsus, "*si*

*vehemens febris urget, in ipso impetu ejus sanguinem mittere, hominem jugulare est.*" The exhibition of emetic tartar in intermittent fever, is, as far as we know, new: and we hope that the commendation bestowed on it by our Danish brethren will not fail to attract attention to it.

*Excellent Effect of a Blister in allaying a violent Chronic Hiccough; by Dr. De Meza of Helsingör.*

A corpulent, plethoric man, ætat. 60, of sanguineo-choleric temperament, accustomed to three meals daily, and much addicted to Bacchanalian pleasures,—after suffering nine days from a disordered stomach, was at length induced to take an emetic. It had operated five or six times, when he was seized with a violent, painful, and unceasing hiccough. I ordered a purgative, and afterwards an antispasmodic clyster, composod of *Assafœtida, Laudan. liq. Sydenham,* and a decoction of groats; I also directed rags steeped in a pint of camphor to be applied every half hour over the abdomen, and twenty-five drops of laudanum to be taken at night. At my visit on the following morning, I found him in the same state, and was informed that the hiccough had continued all night, and prevented him from sleeping. The pulse being full and hard, and the face flushed, I bled him, and directed twelve leeches to be applied to the abdomen; the following medicine was prescribed:

℞. Mosch. orient. gr. x.

Aq. menth. pip ℥viij.

Syrup. Diacodii (papav. caps.) ℥ss. M.

Sumat cochleare unum omni horâ.

In the evening, little alteration; the hiccough was still incessant, though attended with less pain. I now ordered 15 drops of cajeput oil, and the same quantity of Dippel's animal oil, to be taken in oat-soup (water gruel?) every two hours, alternately with the musk mixture; and at night a repetition of the opiate on the following day, still no change; the patient had slept a little, but his sleep was every minute broken by the hiccough. I applied a large blister upon the abdomen, which in the evening had risen well; from this moment the hiccough entirely ceased, and in a few days, the man was able to attend to his business as usual.

*Case of Empyema of the Left Pleural Cavity, attended by peculiar circumstances. Communicated to the Royal Medical Society of Copenhagen, by Svend Svendsen, Army Surgeon.*

A musketeer of the second regiment of infantry, about 20 years of age, and of robust frame, was received into the garrison hospital of this city on the 25th of February, 1821, on account of an inflammation of the lungs. He complained of pain extending over the entire left side, was subjected to the usual treatment of bleeding, blistering, camphor mixture with saltpetre, &c. and on the 5th of May following was dismissed perfectly cured. On the 17th, he again presented himself with a fluctuating swelling on the left side of the chest. It was situated between the sixth and seventh true ribs, near their anterior extremities, and had every appearance of a simple superficial abscess. An emollient poultice was applied, and on the following day, the swelling was punctured; at first the discharge of matter was trifling, but the quantity gradually augmented. The puncture being small, I attempted several times to introduce a small canula, but no sooner had I effected this object than the patient was seized with spasms in the chest, and groaned so piteously, that I immediately withdrew the instrument. I now contented myself with making the opening somewhat wider. The pus evacuated appeared of the laudable kind, and its quantity, gradually increasing, was at length very considerable. By degrees, and despite of our remedies, symptoms of *phthisis purulenta* presented themselves, and the patient died in the last stage of marasmus on the 9th of March, 1822.

*Autopsy.* The heart was shrunk, very small, and in nearly every point of its surface firmly adhering to the pericardium. All that was visible of the left lung was a piece at the root, about two fingers long, and the left cavity of the pleura was filled with pus.

The opening by which the matter had escaped, might admit the blunt end of a common probe; it passed through the parietes of the chest, between the sixth and seventh true ribs, near their anterior extremities, right into the thoracic cavity. Beneath this, the rib was somewhat carious. The liver was unusually firm and voluminous; it projected high into the chest, and covered by the diaphragm, lay immediately over the opening, and closed it, so that on introducing a probe through the latter, the instrument inpinged directly upon the diaphragm resting on the liver, and could not be passed farther unless the diaphragm and liver were forcibly pressed from below."

With respect to this interesting and, we may add, important case, M. Svendsen makes some observations, the justness of which we cannot but regard as problematical. He observes — "although the patient, to judge from his appearance and his own declaration, was perfectly well on the fifth of May, 1821, yet it is more than probable that he then had a *vomica* in the substance of the lung, and that by the continuance of the suppurative process, that organ was at length consumed, and an empyema produced."

We consider this an incorrect view of the origin of the purulent fluid, 1st, because we do not believe that a man in whom an inflammation of the lungs had terminated in the formation of an abscess, (a termination of pneumonia, let it be recollected, of exceedingly rare occurrence) either would appear or would feel perfectly well. And, secondly, the morbid appearances were certainly not such as would have been presented had the lung been consumed by suppuration; but, on the other hand, they were precisely such as are constantly observed in the bodies of those who have died of chronic pleurisy, and we cannot help suspecting that the empyema of the case in question originated from this cause. Again, we greatly object to the word *vomica;* it is a loose and ambiguous term, savouring of those darkened times when Laennec had not yet appeared, and which, under our present enlightened pathological views of thoracic diseases, is, happily, well nigh exploded. We observe also that M. Svendsen states that the patient died under symptoms of *phthisis purulenta;* here again we must charge him with employing a loose and ambiguous expression, for phthisis purulenta, is, strictly speaking, that stage of phthisis when the pulmonary tubercles are in a softened state, while it is very clear that the case related by him was not one of tubercular phthisis. In short—and this is the point to which we wished to arrive—it must be confessed that M. Svendsen betrays a lamentable ignorance of the great and important additions which, in late times, have been made to our knowledge of thoracic diseases. It may afford some consolation to that gentleman, should these strictures by chance meet his eye, to reflect that not only in his own country, but in Germany also, the medical men are alike obnoxious to the same accusation. This is a melancholy fact. Their's, indeed, and not our's, the loss; the German public and not the English are the sufferers; yet are the feelings of universal philanthropy warm in our bosom, and much would it gratify us, and happy should we be, could it be proved that we have arraigned the innocent; but greatly we fear this cannot be.

But to return to our case which, as we before remarked, is an interesting and important one. It places in a striking point of view the danger of performing the operation for empyema without a careful investigation of the case. It is evident that had paracentesis been carelessly performed in the present instance, the diaphragm and liver would have been seriously wounded, and the object, that of evacuating the fluid, unattained. The convulsions and acute pain experienced by the patient on introducing the trocar were clearly referrible, M. Svendsen remarks, to the extremity of the instrument pressing on the diaphragm and subjacent liver.

---

### SOME REMARKS ON MALFORMATIONS OF THE INTERNAL EAR, BEING THE RESULT OF POST MORTEM INVESTIGATIONS PERFORMED IN FIVE CASES OF CONGENITAL DEAFNESS. By Mr. EDWARD COCK, Demonstrator of Anatomy at Guy's Hospital.*

Mr. Cock laments, and laments with reason, the imperfection of our knowledge of the morbid changes of the internal ear. The patience required, and the difficulty experienced in the dissection of that minute organ enclosed in solid bone, are the obvious causes of the absence of research, and the ignorance which has resulted from it.

"When structures, says Mr. Cock, so numerous and diversified, are found assembled within a small portion of bone of compact texture and ivory hardness, —when bones, ligaments, joints, muscles, membranes, secreting tissues, vessels, nerves, and fluids are compressed within so small a compass,—it may be conceived how much time and labour must be expended, and how many ears must be dissected, before we can gain even a superficial and imperfect knowledge of this organ in its healthy state, and how difficult to appreciate every morbid change, or every congenital malformation which possibly may occur in its structures.

Under these circumstances, it is not surprising that congenital deafness should have been almost universally ascribed to paralysis of the auditory nerve, although I believe there is scarcely a case upon record in which the nerve has been found altered in its size or texture, unless through the agency of tubercles, hydatids, or some other cause producing mechanical pressure or lesion of its substance."

Saunders relates one case illustrative of the cause of congenital deafness. In it the labyrinth was occupied by a soft cheesy substance.

Itard, who has published a voluminous work on the diseases of the ear, mentions two cases of congenital deafness, in which the tympanum was filled with a calcareous deposit; also two others, in which a morbid growth had taken place from the membrane lining that cavity, "Végétations produites par la membrane qui la tapise;" and a fifth, where a gelatinous secretion occupied not only the tympanum, but also the canals of the labyrinth. He likewise speaks of a child, where the auditory nerve was converted into a substance resembling mucus, and of a man, in whom it was shrivelled up and reduced to a mere thread.

In Pinel's dissections the cavities of the labyrinth were dry and empty; but these seem to have been cases where deafness occurred in after-life. There are also cases of congenital deafness being caused by an extension of the true skin over the membrana tympani, by the presence of polypi in the meatus externus, &c.

Mr. Cock has within the last two years examined the temporal bones of five children who died in the Asylum for the Deaf and Dumb. In two of these he has detected very palpable deviations from the normal structure. We cannot condense much the ensuing details.

"The subjects examined were all children who died of strumous diseases of the thoracic and abdominal viscera. In three instances, one or both ears were the seat of scrofulous ulceration, affecting the tympanum and meatus externus, with partial destruction of the membrana tympani. In one case, the cavity of the tympanum, together with the mastoid cells, was completely filled with the thick cheesy deposit of scrofula, whilst a similar affection pervaded the whole cancellated structure of the petrous bone. The connexions of the ossicula auditûs were destroyed, but the bones themselves remained entire. I merely mention these facts as indicating the strumous habit of body, which I believe prevails very generally among the deaf and dumb; for as these affections could have existed but for a short time previous to death, they can hardly be supposed to have had any connexion with the congenital defect in the organ of hearing.

* Med. Chir. Trans. Vol. XIX.

I may also remark, that in all the cases examined, the petrous portions of the temporal bones exhibited more than the usual varieties of size and shape. In some the bone was so deficient in particular spots as barely to cover the internal cavities, whilst in others there appeared a preternatural osseous development. In one instance, the petrous bone of a child twelve years old, exceeded in size, hardness, and compactness of structure, that of any adult which I have witnessed.

The malformation which I discovered in two instances, may be described in a few words. It consisted in a partial deficiency of two of the semi-circular canals. The extremities of these tubes opening into the vestibule were perfect, but the central portions were impervious, or rather did not exist at all. In the first case, I had the opportunity of examining the ear from one side only. The vertical and oblique semi-circular canals were both impervious at their central portions."

In the second case both ears were examined. On the right side, the middle portions of the oblique and vertical canals were wanting, the bone presenting an appearance like that already described. On the left side, the horizontal and vertical canals exhibited a similar imperfection. The scala tympani likewise was terminated, at its larger extremity, by a bony septum, which separated it from the tympanum, and occupied the situation of the membrane of the fenestra rotunda.

Independently of these malformations and the scrofulous affections of the tympanum, there were no appreciable alterations in any of the five subjects examined. The auditory nerve seemed sound, as did the chorda tympani.

" In addition to these two cases of mal-formation I may state a third, which was dissected by my friend Mr. Dalrymple, and is now in his possession. In this instance, the aqueduct of the vestibule was so large as to admit the passage of a small probe, whereas, in the natural state, a fine hair can with difficulty be introduced into the canal."

Mr. Cock observes very properly, that as we do not know the peculiar functions performed by the various parts of the organ of hearing, we cannot determine precisely the effects of any individual alteration of them. But there cannot be a doubt that an important part is played by the semicircular canals, as we find them developed fully in many of the lower animals, in whom the tympanum and cochlea are wanting, or exist only as rudimentary appendages.

" The earliest formation of an acoustic apparatus is found in the crustaceous animals, and consists of a membranous sac filled with fluid, and containing a little bone or some cretaceous matter, on which the auditory nerve becomes distributed. Such a structure is seen in the crab and lobster, and appears to correspond with the vestibule of the mammalia.

If we ascend the scale of organization, the next class, fishes, present a development not only of vestibule, but of three semicircular canals, which in some of the tribes are very large.

The reptiles are furnished with an organ in most respects similar to the fishes, but in some of them, a faint trace of a rudimentary cochlea and tympanum becomes apparent.

The succeeding class, or the birds, have the vestibule and semicircular canals perfectly developed; they likewise possess a cochlea approaching in its form to the spiral canals which are found in the highest orders, together with a tympanum and external meatus.

Lastly, the class mammalia exhibits, with slight modifications, the perfect development and elaborate construction which characterize the ear of man."

Thus the vestibule and semicircular canals appear to be the most essential portions of the apparatus for the appreciation of sounds. In the form of an appendix, Mr. Cock adds another dissection of a child that died at the Asylum.

Not a vestige was to be found of the fenestra rotunda on either side, the usual situation of the membrane being occupied by solid bone. The temporal bones were exceedingly large, although soft and spongy in texture. The cavities were more than usually capacious, and the Eustachian tubes presented a remarkable development, being three or four times larger than common. On one side, the aqueduct of the vestibule readily allowed the passage of a large bristle; on the other side, the canal could not be traced through the bone, although its two extremities were more than usually expanded. Suppuration had taken place in one tympanum.

Mr. Cock remarks that:—

" The effect of such a mal-formation would probably be, to prevent the vibratory impression received on the membrane of the fenestra ovalis from being propagated through the vestibule and the canals of the cochlea; for if we consider the labyrinth of the ear as a long osseous tube, commencing at the fenestra ovalis, and terminating at the fenestra rotunda, (and thus closed at both extremities by membrane,) then if a solid structure be substituted for the yielding material, which, in the natural state, closes one extremity of the canal, viz., the round opening, the motion imparted by the ossicula to the membrane of the fenestra ovalis, would no longer produce that undulation of the fluid through the labyrinth, which appears essential to the appreciation of sound."

### Examination of the Organs of Hearing, from the Body of a Boy, aged 13 Years, who had been the subject of Congenital Deafness. By J. Thurnam, Esq.

Passing over appearances which were not morbid, we arrive at the condition of the labyrinth of the right side. It was examined by making a section of the petrous bone with a saw.

" The cochlea, thus divided, was seen to be filled with a matter, which, from its appearance, might have been denominated 'caseous.' This, however, I am convinced, merely arose from the minute saw-dust of the hard bone, having formed a kind of paste by admixture with the aqua labyrinthi. The free edge of the lamina spiralis possessed a highly vascular appearance. The periosteum lining the vestibule and semicircular canals, had evident traces of vascularity, though perhaps scarcely more than may be esteemed as normal; it was lubricated by its proper aqua labyrinthi. The horizontal semicircular canal was imperfect on this side, in about the outer third of its extent; but what, perhaps, might be regarded as an abortive attempt at its formation, existed, as is attempted to be shewn. I did not discover any trace of sacculus or uticulus vestibuli, or of membranous semicircular canals. From the soft state of the acoustic nerve, it was impossible to make out the points of its distribution.

In order to avoid the probable source of fallacy alluded to, I did not employ the saw in the examination of the labyrinth of the left side, but, by means of a strong knife, divided the petrous portion of the bone with a single stroke of the hammer. I thus obtained a view of the vestibule, in which, near the opening of the scala vestibuli, and extending into that canal, there was a minute quantity of a calcareous-like incrustation, closely adhering to the periosteum, and which probably was the remains or rudiment of the 'otolithe' of Breschet. Here, likewise, no uticle or sacculus was met with; there however were very minute, gelatiniform, membranous, semicircular canals. In the cochlea of this side, there was no appearance of anything like a caseous deposit. The osseous semicircular canals were complete on this side."

Mr. Thurnam is not disposed to insist much on the apparent absence of the greater part of the membranous labyrinths, as in the sawing or cutting they might have been partially removed. As Mr. Thurnam remarks, it is yet doubtful what degree of consequence can be attributed to the malformation of the semicircular canals in *one* ear. To us this is rather staggering, and throws doubt on the dependence of congenital deafness on the alterations of these canals. However speculation is at present useless, and the great object is, to accumulate more facts. With one other passage we conclude.

" The existence of calcareous bodies in the vestibular cavity of fishes has been long known to comparative anatomists; but it is only of late, that it has been shewn by Breschet, that these have their analogue in the human species and in all the mammalia, in the form of two minute calcareous bodies, which, accordingly as they are of a hard or soft consistence, he denominates otolithes and otoconies. These bodies, which are situated in the centre of the sacculus and utriculus vestibuli, and appear to be suspended in the aqua labyrinthi, through the medium of minute nervous fibrillæ, have not attracted that attention from British anatomists and physiologists, which, from the interesting nature of the question, might reasonably have been expected; but they deserve to be pointed out here, as liable to mislead the morbid anatomist who is unacquained with their existence. Itard gives a case of congenital defect of hearing, in which, the presence of small portions of calcareous matter in the vestibule and tympanum, is alleged as the cause of deafness. Whether or not the semicircular canals were examined does not appear."

The contributions of Mr. Cock and Mr. Thurnam are interesting and valuable, and we are sure the profession will be under obligations to both, if they pursue their pathological investigations of the state of the internal ear.

---

### Mr. Smith on some Cases in Surgery.

Mr. Smith, Professor of Surgery in the University of Maryland, has related some interesting surgical cases in the North American Archives of Medical and Surgical Science, a transatlantic monthly contemporary. We shall notice such of the cases as seem to contain any practical information.

Case 1.—*Calculus in the Bladder—Sloughing of the Glans Penis.*

A seaman, æt. 71, was admitted into the Baltimore Infirmary, with severe symptoms of stone in the bladder. The urine presented nearly its usual appearance. The patient seemed robust, but had a considerable degree of gastric irritation and febrile excitement. He had laboured under the symptoms of calculus for one year only. On sounding, Mr. Smith discovered one stone rather larger than an egg. The general symptoms forbade an immediate operation. General means were, therefore, adopted at first, and the patient improved to such a degree, that the day of operation was fixed. On that very day, the patient was seized with rigor, succeeded by severe pyrexia. There was also great restlessness—extreme and frequent distress in voiding his water—pain in the region of the bladder—tenesmus—great pain in the glans penis. Bleeding, aperients, and an anodyne were the means resorted to. The pain in the glans penis, however, continued to be extremely distressing, and on the third day after that appointed for the operation, without any obvious preceding inflammation, gangrene displayed itself in the glans about the orifice of the urethra. His pulse about this time suddenly sank, and indeed all the symptoms of approaching dissolution supervened. The prompt employment of stimulants and tonics had very little influence in sustaining the powers of life. He died on the day following.

*Dissection.* The entire glans and a portion of the body of the penis were gangrenous; but none of the ordinary evidences of inflammation were present in the organ. On laying open the abdomen, no traces of inflammation were discoverable in the peritoneum—not even where reflected over the bladder. This organ was somewhat thickened, and, when laid open, its muscular coat was found hypertrophied. The mucous membrane was a little thickened, and that was all. The third lobe of the prostate gland was enlarged, and projected into the cavity of the bladder of the size of a large chesnut. It was soft, and exhibited no appearance of specific disease. In the cavity of the bladder were two calculi, one somewhat larger than an egg, the other very small. They are described as being composed of the phosphatic salts.

The kidneys were very soft and flabby, and mottled on their external surface. The stomach and intestines were slightly inflamed.

Mr. Smith's explanation of the preceding case is as follows:—

"I think it manifest, from the appearances above related, that this man died from irritation inflicted upon the bladder by the calculus; but that which is interesting and important in the case is, that the principal disorganizing effects of this irritation were not felt in the bladder itself, but were, by continuous sympathy, transferred to the glans penis."

We think, with Mr. Smith, that the calculi in the bladder were the principal, though not the immediate cause of the patient's death. Mr. Smith thought it unnecessary to examine the chest. We do not agree with him in that sentiment. Patients with organic disease in any part, are very apt to be suddenly cut off by visceral inflammations, especially by low pneumonia. We see in the dissection no adequate explanation of the rigors and pyrexia with which the patient was attacked. It is not surprising that the glans penis should have mortified. In old persons, whose powers are enfeebled, sloughing of some external part is not uncommon. We have, on several occasions, seen sloughing of the scrotum occur, under circumstances of this description, and we dare say Mr. Smith's venesection helped on the sloughing of the glans in this instance. It is dangerous to bleed old persons for pyrectic symptoms.

CASE 2.—*Large Stones broken in the Bladder.*

Mr. Smith relates a not uninteresting case of two large calculi in the bladder. They were too large to extract through any moderate incision. He, therefore, crushed them. This part of the case is all that requires insertion. We shall give it in the words of the narrator and operator.

"The calculus which I felt, was manifestly too large to be extracted entire through an incision of prudent dimensions. I immediately endeavoured with the forceps to crush it. Anticipating this difficulty, I had provided myself with strong forceps for this purpose. But these I had no occasion to employ, as I had no difficulty in breaking the stone with an instrument of small size. Having done this, I extracted fragment after fragment, some of size equal to that of an ordinary calculus. While effecting the removal of these, I had supposed that I was encountering only one calculus, but it subsequently appeared that there were two of nearly equal, and very large size. The one which I first seized was directly in front of the other, which last was partially encysted; the posterior, upper, and left portion of the bladder being pursed around it. Having removed the fragments of the first, I felt the other firmly embraced by the bladder. It was at first scarcely accessible to the finger, and with difficulty approached by the forceps. At this moment I received very important assistance from my friend Dr. Whitridge, who, pressing with the fingers of both hands, immediately above the pubis, forced down into the vicinity of the wound that portion of the bladder in which the stone was embedded, and made it completely accessible to the finger, so that by repeated efforts I disengaged it from its confined situation, and was then enabled to seize and crush it with the forceps. I then continued to extract fragment after fragment, till I had nearly filled both hands of one of my pupils who received them. In the extraction of these fragments, I still received important assistance from Dr. W. who pressed downward the bladder so as to bring its whole cavity within my reach. Some of the smaller fragments I rolled out from the bladder with my finger, co-operating with my left index in the rectum. At length I could touch with the finger no more earthy substance in the bladder, and on introducing a straight sound through the wound into the bladder, and carefully exploring the whole cavity of the organ, I became satisfied that I had removed every foreign substance from the bladder."

The patient had done remarkably well up to the 12th day, when the report was forwarded. Mr. Smith did not wash out the bladder, because he thinks that operation not only unnecessary, but injurious; the force of the syringe being apt to inject the cellular tissue.

Case 3.—*Lithotomy—Secondary Hæmorrhage.*

On the 26th of November, 1834, Mr. Smith performed the lateral operation of lithotomy on a patient, aged 37. He had laboured under symptoms of stone for three years, but had been treated with bougies for stricture, prior to Mr. Smith's attendance. He was in pretty good health, and presented no evidence of disease of the bladder. There was some little difficulty in the extraction of the stone, which was lodged in the bas-fond of the bladder. The calculus weighed little less than two ounces, and had nearly the size of a hen's egg. There was no more bleeding than usual at the time of the operation, and what blood did flow was venous.

"On visiting him at 4 o'clock (the operation was performed at 12, m.) I found him still suffering but little; yet I felt some anxiety when I discovered that but little if any urine had flowed from the wound. On applying the hand to the pubic region of the abdomen, it was manifest that there was a slight degree of tumefaction there, and there was not a little tenderness. Still there was no urgent necessity for interference.

At 7 o'clock, a message from my patient informed me that he had been just then seized with severe pains and violent straining, which recurred every few minutes, and with increasing violence. I was not for a moment at a loss to account for these distressing symptoms, even before I reached my patient. It was manifest that the flow of urine by the wound had been obstructed by the lodgment of a coagulum, and that the bladder was now labouring ineffectually to relieve itself. Surgeons are well aware that such an occurrence is not unfrequent after lithotomy, and that prompt interference is necessary to give relief.

I provided myself with a straight canula, with large eyes like those of a catheter, and a little larger in diameter than that instrument. This I introduced along the wound into the bladder, which it reached but with little difficulty. It did not appear to encounter in its passage any very firm coagula of blood. On its entering the bladder, there was an instant gush, through the canula, of bloody urine, a considerable quantity of which had accumulated. No sooner had the urine begun to flow, than the patient obtained complete relief, and in a few minutes he became perfectly tranquil. It was manifest from the appearance of the fluid that no recent blood had been poured into it."

Mr. Smith left the instrument in the wound during the night, and before morning the urine that flowed through it was colourless. The instrument however slipped out of the bladder, and in the morning it was necessary to replace it. In the course of a few hours it was withdrawn altogether. The patient now presented no unpleasant symptoms. On the 4th day, he complained of tightness in the forehead, and there was some pyrexia.

On the evening of the 5th day arterial hæmorrhage occurred to some extent. The bladder becoming blocked up with coagula, the canula was introduced. It too soon became obstructed. With a common syringe, Mr. S. now threw cold water through the canula into the bladder, in a brisk stream, endeavouring thereby to break up and dislodge the coagula there formed, and by the influence of cold to constringe the bleeding vessels. Mr. S. continued to repeat the injection until at length the water returned nearly colourless, and it was manifest there was no longer any effusion of blood. Soon after, he administered an enema, by which an evacuation of rather hard fæces was obtained from the bowels. The patient again grew comfortable, and the water flowed from the wound without impediment.

Mr. Smith concluded that the bleeding proceeded from small rather than large vessels, for reasons sufficiently obvious. All went on pretty well till the night of the 8th, when Mr. S. was again summoned.

"On reaching him, I found him suffering from the irritation caused by the accumulation of coagula in the bladder, and also from loss of blood. There were frequent and ineffectual contractions of the bladder and abdominal muscles—there was some degree of tumor in the pubic region, and much tenderness—the pulse was one hundred and forty in the minute, quick and feeble—the countenance was exanguious and anxious—he was restless. No recent blood had yet flowed externally, but coagula had been discharged from the wound. I immediately introduced the canula and emptied the bladder of the fluid blood and water which it contained. I then prepared a strong solution of alum, which, with the syringe, I threw into the bladder, and then allowed its reflux through the canula. In order that the astringent liquid might certainly come in contact with the bleeding vessels, I then injected the bladder, and quickly withdrawing the canula, allowed the liquid to escape along the wound. I then introduced the canula so far into the wound, as that I supposed its eyes to be engaged in the wound of the prostate, and repeated the injection, the liquid returning by the wound. The Tinct. Digital. with a solution of Nitr. Potass. was now directed.

These means, which were attended with but little suffering to the patient, effected the immediate suppression of the hemorrhage, nor did it afterwards recur."

But, though the bleeding did not recur, the patient remained in an alarming condition. The pulse was very frequent—there were irregular determinations of blood to the surface—the head was hot, and affected with a sense of constriction across the brows. We need not pursue the treatment or subsequent details. On the evening of the 11th day, the patient became affected with symptoms of acute pleurisy, or pericarditis—the constitutional symptoms being a compound of those of irritation and exhaustion. These symptoms were somewhat mitigated, and no more, by cautious bleeding, and remedies of that description; but, on the night of the 18th day, the febrile symptoms were all aggravated, and delirium, with intolerance of light &c. were added. On the night of the 20th day, he died. No inspection after death was permitted.

We relate this case because it is practically interesting. Mr. Smith seems astonished that the patient should die, and appears puzzled on what to lay the blame. Extended experience in the operation of lithotomy will convince him that, do what he may, some patients *will* die; some are cut off by one thing, some by another, but still some *will* die. Secondary hæmorrhage after the operation is not common, but yet it occasionally takes place. It is a very serious occurrence. We have only seen one instance out of four or five, in which it did not prove fatal. That instance was in the case of a child. Primary hæmorrhage also, we mean hæmorrhage within the first few hours, is always to be looked on with misgiving. The patients in whom it happens generally do ill. Either they get diffuse inflammation of the pelvic cellular tissue, or circumscribed abscess in the pelvis, or some bad consequence or other takes place. In hæmorrhage after lithotomy, the cellular tissue is much disturbed—the blood is driven into it—the urine follows—and the surgeon is obliged to poke about in it with canulæ, or sponge, or lint, or his finger, or something. Certain it is, that the patients far too frequently do ill.

Mr. Smith makes a remark that proves him to be a practical man. He observes that a persistence of a frequent weak pulse is alarming. Nothing can be more true than that, after an operation or an injury, the continuance of a weak and frequent pulse, and a skin slightly warm, is a symptom ominous of coming mischief. Such patients usually get cellular inflammation, or secondary inflammation of the viscera. Let the surgeon always look to it.

The following is an interesting case of aneurismal varyx—an aneurism resulting from puncture of the artery in bleeding, the aneurism consisting of a sac formed out of cellular tissue, intervening between the artery and vein, and communicating with both.

Case 4.—*Aneurismal Varyx—Ligature of the Brachial Artery.*

A. B. a coloured man, æt. 26, was sent to Mr. Smith, on the 25th of Nov. for an aneurism at the bend of the arm.

"The disease had resulted from an accident in bleeding, which had occurred in the hands of a gentleman remarkable for the neatness with which he usually peforms phlebotomy. The accident was owing to the local relations of the parts concerned being remarkably different from those which usually exist. It was the median-cephalic vein which had been opened, but this vessel lay much lower than is usual—that is, nearer to the inner condyle, while the median basilic was very short. The brachial artery, on the other hand, lay nearer than usual to the radial border of the arm. We entertain but little apprehension of wounding the brachial artery when we strike the median-cephalic vein, and this undoubtedly was the cause of the occurrence in this instance. The artery was wounded through the vein and directly beneath it. The blood gushed rapidly at the time, but its flow was soon embarrassed in consequence of the formation of a thrombus. A compress was finally applied, and, although bleeding in small quantity occurred several times, yet the hemorrhage was never serious. The part was a good deal swelled at the time, and painful. At length the inflammation disappeared, and there remained a pulsating tumor. At the time it was first examined by me, it had the magnitude of the half of an egg, and had a distinctly circumscribed cyst. The vein was evidently concerned in the tumor, but to what extent the tunics of the vein entered into the walls of the aneurism, it was by no means easy to determine. It appeared to me, however, on making a most careful examination, that the principal part of the cyst was formed in the cellular tissue, intervening between the artery and the vein—that the inner orifice of the wound in the vein had become expanded, and its margin incorporated with the cellular walls of the aneurism.

The peculiar aneurismal thrill was very manifest in the pulsation of the tumor. At each throb the blood was forcibly injected into the vein, suddenly expanding it both beneath and above the tumor. Beneath, however, this swelling of the vein extended no further than to the first valve, which was half an inch from the tumor. Above, the expansion of the vein was obvious half way up the arm, and the thrilling rush of the blood was distinctly felt at each pulsation of the heart. Pressure upon the brachial artery, above the tumor, arrested the pulsation. Pressure upon the tumor emptied it of its contents. The walls of the tumor toward the surface were thin.

The compress having been already ineffectually employed in this case, and the tumor obviously increasing, I immediately advised that the ligature of the brachial artery should be performed without delay. For this operation he repaired to the Baltimore Infirmary, where I was then officiating as surgeon. Preparatory to the operation, I caused blood to be taken from the arm—prescribed a low diet and repose for two or three days.

The operation was performed in the presence of the medical class of the University of Maryland, and with the assistance of the pupils of the house. The patient was seated in a chair, and his arm was extended upon a table. The incision was made as usual along the border of the biceps, the fascia of the arm being laid bare at the first stroke of the knife, and opened by the second. On introducing the finger into the wound, I felt the pulsations of two arteries—the one in the usual position of the brachial, close beneath the border of the biceps—the other toward the ulnar border of the arm, but not remote from it. On compressing the latter, the pulsations of the tumor did not cease, but when this was practised upon the former, they were instantly commanded. The pulsations of these vessels were so equally strong, that I immediately inferred that, in this instance, the division of the brachial had occurred at a higher point than usual, and that only the radial branch was concerned in the operation. With this impression, I immediately applied the ligature to the artery beneath the biceps. The pulsations of the tumor at once ceased, its volume diminished, and it became flaccid. A silk ligature was employed, one extremity of which was left hanging from the wound."

This was dressed with adhesive plaster, &c. Next day the tumor was found to pulsate slightly, and there was a good pulsation in the radial artery, at the wrist. The brachial artery, also, pulsated feebly on its entrance into the tumor, but no vessel on the ulnar side of the arm could be distinguished, nor was the pulse in the ulnar, at the wrist, any stronger than that of the radial there. Mr. S. therefore supposes that the collateral vessel he felt during the operation was the anastomotic, greatly enlarged. On the third day, there was no pulsation in the tumor. On the fifth day it had become hard and incompressible. On the 13th day, the ligature came away. A slight degree of inflammatory action subsequently occurred about the tumor, but it quickly passed away. The tumor was now no more than half the size it had been prior to the operation.

The preceding cases are facts not unworthy of perusal.

---

REMARKABLE DISEASE OF THE BRAIN, ATTENDED WITH DISTRESSING SYMPTOMS. By JAMES JOHNSON, M.D.

The subject of this melancholy case was an eminent artist residing in Albemarle-street. The complaint commenced several years ago, in the form of flashes of light before the eyes—and to these were afterwards added, pains in the head, and diminished distinctness of vision. This last symptom gradually increased till his sight was totally destroyed. The grand phenomenon, however, which chiefly annoyed this unfortunate gentleman, was a series of the most dazzling images, perpetually playing on the optical apparatus, by day and by night. Their brightness was unspeakably distressing. Sometimes they would assume the forms of angels with flaming swords, every motion of which seemed, like an electric flash, to blind the eye and sear the brain by the intensity of its light. The forms and shades, however, of these spectral images were perpetually changing, but without any mitigation of the sufferings which they produced. With the exception of some irritability of temper, there was not the slightest affection of the intellectual powers. The memory, the imagination, the judgment were unimpaired. He was led about the streets by one of his servants; and he attended to all matters where his sight was not engaged, with the greatest punctuality. The eyes themselves presented no appearance of disease.

The symptoms above-mentioned were mitigated, from time to time, by tartar-emetic plasters to the nape of the neck, leeches to the temples, aperient and diuretic medicines. In the Spring of 1835, however, he was seized with all the usual symptoms of apoplexy. He lay in bed motionless and insensible, passing the urine and fæces involuntarily—the pupils dilated—and the power of speech gone. To the astonishment of his medical attendants, he rallied from this state, whether from the powers of nature, or active depletion, may be doubtful; and, after a few weeks, he was able to walk to the City, and transact business as usual! But the spectral images, of dazzling and exquisitely painful brightness returned, with, if possible, increased intensity.

In the month of August, he was suddenly seized again with the apoplectic symptoms above-mentioned, and notwithstanding the same means were employed as on the former occasion, he died at the end of three or four days from the commencement of the apoplectic invasion.

*Autopsy.* The body was examined on the day after his death. There was nothing unusual in the membranes of the brain. The right lateral ventricle contained nearly two ounces of clear fluid. The left ventricle was occupied by a series of hydatid-like cysts of various sizes, and filled with fluids of various consistencies and colours. This cluster sprung from the floor of the ventricle, by a kind of peduncle, and penetrated into every sinuosity of the cavity, pushing its branches anteriorly, so as to pass over and before, the thalamus nervi optici of that side, and even into the opposite hemisphere of the brain, destroying all the parts in its march. Both thalami were reduced to a pulp, as was, indeed the whole of the anterior lobes of the brain, which would scarcely bear the slightest handling, without falling into a state of deliquescence. The optic nerves were pressed upon by the cystic or hydatid mass, and reduced to little more than the size of threads, and these of very soft consistence. There was no change in the coats or humours of the eye.

The most remarkable phenomenon in the above melancholy case, is the intensity of brightness which always accompanied the spectral images. Whatever were their shapes, the dazzling and painful splendour never forsook them. It rendered his life a scene of dreadful suffering for some years.

It is scarcely less remarkable that the intellectual faculties should have remained entire, while the anterior lobes of the brain were undergoing the process of softening which they displayed on dissection! Or did this *ramollissement* take place during the three or four days of apoplexy prior to death? If it existed long before the fatal event, phrenology will have some difficulty in accounting for the integrity of the intellectual faculties up to the apoplectic seizure. Was the serous effusion into the right ventricle the cause of the apoplexy?—or the consequence of it?—or was it a gradual accumulation, and not mainly instrumental in the final catastrophe? What was the cause of the first attack of apoplexy, and why did he recover from it?

These are questions more easily asked than answered; but the case will suggest several other queries and reflections beyond what are stated above.

---

### Salivation from Iodine.

Dr. Mackall, of Calvert County, Maryland, reports this in the Journal from which a preceding article is taken.

" In Sept. 1834, I was called upon to prescribe for Mrs. W. who was laboring under an enlargement of the spleen. This lady was of what is denominated as bilious temperament, lived in a malarious district, and had, as a matter of course, been subjected to frequent salivation. Two months previous to my being called to her, she had suffered a slight attack of remittent fever; for the cure of which she had been profusely salivated.

When I first saw her, the case presented the following symptoms: her tongue was clean and moist, and except a slight degree of redness around the border, presented no morbid appearance. Her pulse was small, frequent, and irritated; appetite good, bowels regular, and evacuations healthy. Her spleen filled the whole left hypochondrium, and was extremely hard. Under these circumstances I administered the tinct. iodine, in doses of eight drops, thrice a day. In four or five days from the commencement of this course, her tongue became furred; her gnms very sore, and a most profuse ptyalism took place, accompanied with strong mercurial fetor. The use of the iodine was suspended for two weeks, in which time the ptyalism ceased entirely. In a few days I resumed the use of the remedy, with the same result; but in a very much slighter degree.

The opinion which I formed at the time of the case, was, that the iodine, by increasing the activity of the absorbents, had caused a portion of mercury to be taken up, which had been lying dormant. And I still think that the ptyalism could have been produced only by the absorption of mercury."

However this may be, it is certain that several of the metals, besides mercury, will occasionally produce salivation. Gold is said to do it; arsenic we have seen do it; and iodine has been also reported a sialagogue. We cannot say that we have witnessed such effects from it; but we cannot doubt that now and then they do take place.

We would make a remark, which, practically, we think will not be found unimportant. When a patient has taken large quantities of mercury, and particularly when he has taken it to the extent of seriously impairing his constitutional strength, small doses will frequently occasion salivation. Nothing is more common than for a poor wretch broken down by mercurial cachexia to be salivated, and severely so, by a few grains of blue-pill. We see this constantly in the Lock Hospital. Let the surgeon beware how he plays with mercury in individuals circumstanced thus.

---

### Identity of Gonorrhœa and Syphilis.

It is utterly astonishing with what pertinacity some men will re-assert what has again and again been denied and disproved. Every body knows the vexata questio of the identity or non-identity of syphilis and gonorrhœa. There are some points certainly on which no man can safely say that the line of demarcation is distinct; but still there are such wide, such important differences between the two maladies, that for practical purposes they cannot be regarded as the same. Yet see through what spectacles a theorist will look.

Professor Hufeland announced thirty years ago that " gonorrhœa arising from infection is always syphilitic, but is modified and rendered less infectious by the secretion poured out by the mucous membrane of the urethra." The Professor has actually the hardihood to assert that this proposition has been confirmed by long experience. Confirmed by long experience! Why it is contradicted by the experience of every man of common sense. What surgeon does not see hundreds of claps, and what surgeon sees secondary symptoms from them? If he sees any they are very few indeed. The believers in the indentity of the two poisons shelter themselves behind the ridiculous assumption, that the poison of syphilis is modified by the mucous discharge, and that gonorrhœa, which is syphilis in the mucous membrane, presents on this account mitigated features. This might have some plausibility if only men existed in the world, and if they alone had venereal diseases. But what shall we say of the ladies? The same membrane in them is the seat of both affections, yet still the essential distinction between them is maintained—syphilis as a general rule requiring mercury, and being disposed to occasion secondary symptoms—gonorrhœa, as a rule, not requiring mercury, and not being disposed to give rise to secondary affections.

The following results of Professor Hufeland's experience are really a practical curiosity.

1. *The same cause.*—Two individuals may be infected by one person,—the one may have gonorrhœa,—the other a syphilitic chancre.

2. *The same effects.*—A person affected with gonorrhœa may communicate syphilis to another, or infect himself with that disease. Gonorrhœa matter introduced into the eye may give rise to a venereal ophthalmia; or a gonorrhœa too suddenly suppressed may be followed by bubo, chancre, condylomata, &c. and daily experience proves, that fluor albus of the female, which supplies the place of gonorrhœa, is capable of occasioning syphilis.

3. *The same treatment.*—Although the professor acknowledges that a majority of cases of gonorrhœa are cured either by the powers of nature, or antiphlogistic treatment; he affirms, that when this is not the case, and pains take place in the urethra, with inflammation in the throat, and other consecutive symptoms, calomel is always the most efficient remedy.

The difference between the virus of the two affections is, that, that of gonorrhœa is incorporated as it were with the mucous secretion, and acquires thereby a milder character, so that it may lose its infectious quality both as regards a second person and the individual himself. It is besides sometimes thrown off with the mucous secretions. The virus of a chancre, on the contrary, is more active and corrosive, for the same reason that corrosive sublimate, or any other poison, introduced into the system in its isolated state, is more energetic than when enveloped in mucus.—*Journal für Praktisch Heilkunde,* 1834.

The combination of assumptions and absurdities in the preceding passages, is absolutely marvellous. Yet the opinions bear the respectable name of Hufeland, and they have been retailed uncriticised in America and France. Some of the reputed facts are downright nonsense. What can the author mean by saying that "a gonorrhœa too suddenly suppressed may be followed by a chancre?" The whole affair is utter twaddle.

---

### On Venereal Affections of the Testis.

Such is the title of a paper in the number of our very excellent contemporary of Dublin for November last. The title in question is a palpable misnomer. Can any thing be more loose than the following passage with which the essay opens.

" The existence of an acute or chronic enlargement of the testis, originating in the action of the venereal poison, has been questioned by several practitioners of eminence, but so many surgeons of the highest authority have admitted the existence of venereal affections of the testicle, that we must assume the case to be proved; but here our knowledge, derived from published opinions of practitioners, ceases."

We cannot at all agree with the author, who, we should observe, is Dr. Cusack, that the existence of acute and chronic enlargement of the testis originating in the action of the *venereal* poison, has been questioned. Gonorrhœa, we presume, is a venereal disease, and nobody doubts that gonorrhœa will occasion, and daily does occasion, both acute and chronic inflammation of the testicle. Dr. Cusack, we presume, alluded to the syphilitic poison, but a lecturer on surgery, as he is, should have used more explicit language. This is, after all, only verbal criticism, yet verbal criticism is sometimes indispensable to prevent liability to misconception.

Dr. Cusack's object is to endeavour to point out the syphilitic affections of the testis. We may state broadly that no good account of these affections exists, indeed we may go farther and remark, that no good description of syphilis exists. The authorities adduced by Dr. Cusack on the subject are these.

1. Sir A. Cooper believes that the testis is most frequently implicated in company with eruptions and periosteal affections. He supposes that the tunica albuginea is the part first engaged, and the internal fibrous and tubular part of the testis secondarily. Yet he never had an opportunity of verifying this by dissection.

2. Mr. Ramsden, who almost doubts the existence of the affection, seems to consider its characteristic marks, to be uniformity and smoothness of surface, combined with globular form, hardness, and resistance.

Dr. Cusack's experience and observations are as follow :—

As the disease, as far as Dr. Cusack has observed, commences in the body of the testis, there is little alteration in the form of the organ in the first instance; as the enlargement advances, the tumor becomes more globular, the epididymis soon becoming involved, and lost in the general mass; the tumor has a fleshy feel, but differs much in density in different parts; it is said to be smooth and uniform on the surface, and primarily it certainly is so generally. Partial adhesions in the cavity of the tunica vaginalis, combined with effusions into that cavity, even independent of the internal changes which may be going on, render this a very uncertain symptom; so uncertain indeed, and so little uniform are the primary appearances, that Dr. Cusack believes the best practitioners would be unable to make a perfect diagnosis if unacquainted with the history of the case, and the attending circumstances.

The termination of the disease is either resolution, or suppuration, or induration, and the formation of granular bodies, ending in total destruction of the functions of the organ. Dr. Cusack hesitates to believe, and is unable to affirm from his own practice, that this affection of the testis is met with in the earlier stages of secondary symptoms, or during the presence of any of the forms of true papular eruption.

The acute form alluded to is met with accompanying venereal hectic, pains in the bones, and either a scaly eruption or perhaps a solitary spot, apparently belonging rather to the genus acné; but these instances are comparatively rare, and of fifty patients at present under treatment in Steeven's Hospital there is not one case to adduce in confirmation of such an opinion. The patients who suffer from this affection are those persons who labour under affections of the periosteum and bones, and bear the marks of having suffered from pustular and tubercular eruptions. The specimens of the disease submitted to the Society were wholly derived from persons whose constitutions were broken down from the protracted forms of the disease with which practitioners are familiar, but neither from his own opportunities nor any other source could Dr. Cusack exhibit a specimen of the changes which take place in the more curable forms of secondary syphilis. Ten preparations (selected from the collection in Park-street) were laid on the table exhibiting the disease in all the stages of its progress, from a small circumscribed tubercle in an otherwise sound testis, to the contracted, indurated, and completely disorganized gland. The structure of the tubercle is rather soft, but harder than common scrofulous tumour, and surrounded by a thickened layer resembling a cyst, the product of inflammatory action. In one preparation the tubercle is in the lower part of the testis, which is otherwise so sound that the epididymis adadmitted of injection by mercury, while in the opposite testis the tubercle was softened, and contained a glairy fluid.

Mr. Cusack noticed that the adhesions of the tunica vaginalis usually took place at the inferior portion of the testis; and also gave it as his opinion, that the granular fungus often succeeded to the suppuration of one of these tubercular chambers.

One reflection must immediately occur to all who are conversant with the diseases of the testis. The tubercular alteration described by Dr. Cusack as constituting the syphilitic affection of the testis, differs in no material respect from the tubercular alteration that constitutes what is commonly known under the name of chronic inflammation of the testicle.

We have seen many instances of syphilitic affection of the testis, and for many reasons we have little doubt that syphilitic affection does not present any very peculiar structural changes. The disease occurs for the most part in what are called cachectic cases—that is in persons with sloughy ulceration of the throat, or rupia, or spreading phagedenic secondary ulcerating eruptions—in persons, in short, who are broken down by syphilis, or mercury, or both. So far as we have seen, the disease *does* appear to commence in the epididymis, or the tunica albuginea. The pain is not usually very severe, the inflammation is seldom acute, the testis is generally hard and irregular, particularly if the disease has existed for any time, and both testicles are often attacked in succession. We said that we did not think there was any striking peculiarity of structural alteration in these cases. It has always seemed to us that the disease was chronic inflammation modified only by the state of system. We have once, and but once had an opportunity of dissecting a testicle under these circumstances. The patient had had sloughing of the fauces—rupia—nodes—and phagedenic ulceration of the glans, which, after removing the greater part of it, terminated, in chronic ulceration and induration of the anterior part of the corpus spongiosum urethræ. Both testes had been frequently attacked with inflammation in conjunction with the appearance of a fresh crop of rupia, or fresh periosteal inflammation. On dissection, the epididymis on each side was greatly thickened and of gristly hardness—the testes were scarcely larger than natural, but irregular, and knobbed. The alteration seemed to consist partly in the deposition of a yellowish, inorganized tubercular matter, and partly in thickening of the tunica albuginea, if not of its fibrous septa.

We have seen the testicle enlarged in connexion with tubercular eruption, unattended with decided cachexia, and uncomplicated with mischief from mercury. In that instance the testis was a good deal larger than natural, uniform on the surface, hard, not very painful. The condition of the epididymis, we do not recollect. So far as the appreciable condition of the testis went, there was nothing to distinguish it from the state of chronic inflammation. The disease subsided under a course of mercury, but so does chronic inflammation.

We have now under our care a case, apparently of syphilitic affection of the testis. The patient is a gentleman who was not aware of the existence of any primary sore. But he became the subject of ulceration of the tonsils, and an eruption resembling in every particular the syphilitic lepra. For a long time sarsaparilla, iodine, and many other remedies were tried without effect. He then was attacked with inflammation, of a sub-acute character, of the right testicle. This produced some effusion in the tunica vaginalis. Then there was a slighter affection of the left testis. He then came under our care. We put him under a mild course of mercury. The disease of the testis was much relieved, the left being apparently restored to its natural condition, although some hardness, and effusion remained on the right side. The ulceration of the throat healed, but the copper-coloured spots did not disappear. We continued the mercurial course as long as we could with prudence, and since that we have exhibited sarsaparilla, tonics, and medicines which we need not particularly mention. There is still, we say, some induration of the right testis, and the eruption has shewn a disposition to pustulate and scab. The case cannot therefore be yet considered as decisive of the cure of syphilitic affection of the testicle. But there are some peculiarities in it, which withdraw it from the range of common syphilitic cases.

The subject is one well worthy of attention.

---

### Phrenology.—Case of Supposed Impairment of the Faculty of Language.

The following short articles are derived from the last number of our quarterly contemporary, the Phrenological Journal.* The first is a case related by Mr. Gibson, Surgeon of Montrose. We notice it because we think some exception may be taken to the light in which it is regarded.

*Case.* In the evening of the 11th of May, Mr. G. was called to a woman, about 30 years of age, who had recently weaned her first child. She had latterly emaciated, and frequently complained of headache. About 8 P.M. she was found partially insensible, with her hand pressed on her forehead, and only able to say that she experienced great pain.

"She was put to bed, and was much in the same state when I saw her. She still often put her hand to her head, groaned and muttered occasionally, and took no notice of what was going on around her, except in firmly resisting me in making an attempt to bleed her. The pulse was not affected, nor was there any heat of head, or of surface generally. We succeeded in forcing into her mouth a little sugar, with three drops of croton-oil upon it, of which she appeared to feel the disagreeable flavour. Next morning she was quite insensible. I then bled her freely, and gave more croton-oil, till the bowels were well acted on. She, however, remained perfectly insensible for five days; during which time leeches were applied to the head, blisters and sinapisms to the nape of the neck, spine, and lower extremities, and turpentine embrocations to the loins and epigastrium. A little tea, which she swallowed from a tea-spoon, was her sole nourishment. Gradually she began to throw off the stupor, to notice, to take nourishment, and to move about. Her speech, however, was very much affected :—At first she only uttered inarticulate sounds ;

* Phrenological Journal, Dec. 1835.

then single words very indistinctly, and generally inapplicably; and, when she did begin to utter sentences, they were very unconnected and unmeaning, the different words being either wrong or strangely jumbled together. It has been very slowly that she has acquired the use of speech, and it is only now that, with difficulty, she can give an account of her feelings during her illness. She says that she was first attacked with pain in one side of the head; that it soon went to her forehead; and that then, as she expresses it, 'it fell down into her EEN,' where it has remained more or less ever since, excepting, of course, during the five days that she was insensible. She refers the pain to a spot immediately above and behind the eyes; and, when I desire her to point out the spot, she puts her fingers beneath the superciliary ridge, presses back the eye as far as she can, and says that it is there and farther back. She complains much of her defect of speech; she says that she knows perfectly what words she ought to use, but cannot get them expressed. She has no other complaint now remaining, excepting a slight dimness of sight, which is going off gradually."

Setting aside phrenological considerations, there can be little doubt from the collateral circumstances, the history, the symptoms, and the results of treatment, that this patient laboured under inflammatory action of the brain in *some* part. Was that part the seat of the faculty of language? To answer this question we must carefully distinguish two things—the faculty of language, that is, the conception and remembrance of conventional signs for ideas—and the power of giving those symbols utterance. The first is obviously an intellectual operation, the province of the brain—the second, a comparatively mechanical process effected by the tongue.

In the case we have related it seems clear to us that it was not the intellectual function that was injured, but the power of directing the tongue that was impaired. The patient said that she knew well enough what words she ought to use, but she could not get them *expressed*. If the conception and remembrance of language had been lost, she would *not* have known what words to use. It is clear that she had lost the due control of the various muscles engaged in the articulations of sound. The truth is, that some of the cerebral nerves were interfered with at their origins. Probably those that suffered most were the ninth and optic; for, independently of the defect of speech, there was slight dimness of sight. We make these remarks because loose observations and indefinite statements are seldom of benefit to any science.

---

### PHRENOLOGICAL QUACKS.

We are glad to perceive that our phrenological contemporary has taken these gentry in hand. It would be disgusting, if it was not so absurd, to witness the mountebank performances of some persons who profess phrenology. They thumb the heads of gaping or of laughing audiences at sixpence or a shilling each, and pronounce, ore rotundo, the elaborate characters of Styles and Noakes, who, fifty to one, have got no characters at all. We have been at some of these exhibitions, and a more complete travestie of a science we never in our lives have seen. We hope the philosophical phrenologists will put this egregious humbug down.

